# Validity and reliability of wearable inertial sensors in healthy adult walking: a systematic review and meta-analysis

**DOI:** 10.1186/s12984-020-00685-3

**Published:** 2020-05-11

**Authors:** Dylan Kobsar, Jesse M. Charlton, Calvin T.F. Tse, Jean-Francois Esculier, Angelo Graffos, Natasha M. Krowchuk, Daniel Thatcher, Michael A. Hunt

**Affiliations:** 1 Department of Kinesiology, McMaster University, Hamilton, ON Canada; 2 Motion Analysis and Biofeedback Laboratory, University of British Columbia, Vancouver, BC Canada; 3 Graduate Programs in Rehabilitation Sciences, University of British Columbia, Vancouver, BC Canada; 4 Department of Physical Therapy, University of British Columbia, Vancouver, BC Canada; 5 The Running Clinic, Lac Beauport, QC Canada

**Keywords:** Inertial sensors, Inertial measurement units, Gait, Biomechanics, Validity, Reliability, Review

## Abstract

**Background:**

Inertial measurement units (IMUs) offer the ability to measure walking gait through a variety of biomechanical outcomes (e.g., spatiotemporal, kinematics, other). Although many studies have assessed their validity and reliability, there remains no quantitive summary of this vast body of literature. Therefore, we aimed to conduct a systematic review and meta-analysis to determine the i) concurrent validity and ii) test-retest reliability of IMUs for measuring biomechanical gait outcomes during level walking in healthy adults.

**Methods:**

Five electronic databases were searched for journal articles assessing the validity or reliability of IMUs during healthy adult walking. Two reviewers screened titles, abstracts, and full texts for studies to be included, before two reviewers examined the methodological quality of all included studies. When sufficient data were present for a given biomechanical outcome, data were meta-analyzed on Pearson correlation coefficients (r) or intraclass correlation coefficients (ICC) for validity and reliability, respectively. Alternatively, qualitative summaries of outcomes were conducted on those that could not be meta-analyzed.

**Results:**

A total of 82 articles, assessing the validity or reliability of over 100 outcomes, were included in this review. Seventeen biomechanical outcomes, primarily spatiotemporal parameters, were meta-analyzed. The validity and reliability of step and stride times were found to be excellent. Similarly, the validity and reliability of step and stride length, as well as swing and stance time, were found to be good to excellent. Alternatively, spatiotemporal parameter variability and symmetry displayed poor to moderate validity and reliability. IMUs were also found to display moderate reliability for the assessment of local dynamic stability during walking. The remaining biomechanical outcomes were qualitatively summarized to provide a variety of recommendations for future IMU research.

**Conclusions:**

The findings of this review demonstrate the excellent validity and reliability of IMUs for mean spatiotemporal parameters during walking, but caution the use of spatiotemporal variability and symmetry metrics without strict protocol. Further, this work tentatively supports the use of IMUs for joint angle measurement and other biomechanical outcomes such as stability, regularity, and segmental accelerations. Unfortunately, the strength of these recommendations are limited based on the lack of high-quality studies for each outcome, with underpowered and/or unjustified sample sizes (sample size median 12; range: 2–95) being the primary limitation.

## Introduction

Gait analyses are important for evaluating movement in healthy and pathological populations by assessing a range of biomechanical outcomes from simple spatiotemporal parameters to complex three-dimensional (3D) joint angles [1, 2]. While laboratory-based, optical motion analysis systems remain the gold standard for gait analysis, they are expensive, resource intensive, and largely immobile, which limits their accessibility in both research and clinical settings [3]. Alternatively, recent technological advancements have led to the growing popularity of more affordable, easy-to-use, and accessible wearable sensors for the analysis of gait patterns [4].

Wearable technology refers to any electronic device that can be worn, but inertial sensors are the most common type of wearable sensor for measuring gait [5]. These sensors apply the principle of inertia to measure linear accelerations (i.e., accelerometers) or angular velocities (i.e., gyroscopes). Independently, inertial sensors can provide information on the motion of segments, or timing of gait events. Further, inertial sensors can be integrated into what is called an inertial measurement unit (IMU), which contains a 3-axis accelerometer and a 3-axis gyroscope, as well as, in some cases, a 3-axis magnetometer to assess heading direction [6]. The fusion of data from these sensors facilitates the assessment of segment orientations and joint angles [6, 7]. Therefore, inertial sensors, either on their own or combined in an IMU, provide an excellent opportunity to collect a variety of valuable and objective outcomes related to gait.

With the increasing popularity of wearable sensors, there have been an increasing number of studies examining their validity and reliability for gait analysis. Similarly, while there are many reviews of wearable sensor literature available, most have taken a descriptive approach to outline potential applications [5, 8] or methods [4, 9–11]. Therefore, there remains a lack of systematic reviews and meta-analyses which synthesize the results of the many validity and reliability studies which have examined inertial sensor outcomes for gait analysis. Recently, two systematic reviews examined 3D joint kinematics from inertial sensors across a variety of movements and populations [12, 13]. While they were unable to quantitatively pool data due to study heterogeneity, they were able to qualitatively suggest sagittal, and to a lesser extent frontal, plane lower limb joint kinematics displayed acceptable validity. Nevertheless, these findings remain confounded across a variety of human movements and populations. Therefore, addressing kinematic outcomes in only healthy adult walking may help to homogenize findings and recommendations. Further, there remains a growing body of literature that addresses a variety of spatiotemporal and other biomechanical outcomes assessed across a variety of locations (e.g., back, shank, foot, etc.) in walking which have yet to be addressed in a systematic and quantitative manner. Addressing this gap in the literature will help future researchers to identify not only the most valid and reliable of these variables, but the optimal placement of sensors to measure them. Therefore, our aim was to conduct a systematic review and meta-analysis to determine the i) concurrent validity and ii) test-retest reliability of IMUs for measuring biomechanical gait outcomes (e.g., spatiotemporal, kinematic, or other) during level over-ground or treadmill walking in healthy adults.

## Methods

### Eligibility criteria

We included journal articles that assessed the validity or reliability of IMUs measuring biomechanical outcomes during walking in healthy adults. For a validity study to be included, it must have assessed the concurrent validity (i.e., simultaneous collection) of inertial sensor measured biomechanical gait outcomes as compared to what we defined to be gold standard devices (See Additional file 1) in healthy adults. Similarly, for a reliability study to be included, it must have assessed the test-retest reliability (i.e., between-day, within-day, or between-tester; involving the same measure/device/placement with removal between sessions) of IMU-measured biomechanical gait outcomes in healthy adult walking. Biomechanical gait outcomes included spatiotemporal parameters (e.g., step time, step length, stance time, etc.), segment or joint kinematics/kinetics, or other biomechanical outcomes (e.g., accelerations, stability, regularity, etc.). However, we did not include per count measures such as gait speed or cadence as these require two components (e.g., time and distance) and can often be measured as an average over the entire dataset. Additional details on our inclusion and exclusion criteria can be found in Additional file 1.

### Study identification and screening

A systematic literature search was conducted with the help of a librarian to identify all relevant journal articles in the following databases: MEDLINE, Embase, CINAHL, Web of Science, and Compendex. Our search criteria were based on the combination of four broad topics: inertial sensors, gait biomechanics, healthy adults, and validity/reliability. Each topic included an expanded set of terms, keywords, and syntax specific to each database to maximize the breadth of our search. A detailed list of our search strategy for each database can be found in Additional file 2. This search was conducted on May 7th, 2019.

Following the removal of duplicate items, titles and abstracts were screened by two independent reviewers (CTFT and DT) to determine their eligibility based on the aforementioned criteria. Studies that were deemed potentially eligible were passed to full-text screening where two independent reviewers (CTFT and DK) conducted a thorough examination of each article to determine if it would be included in our review. Moreover, the reviewers also identified eligible components of the study for future analysis; for example, a study may pass in reliability criteria, but fail validity criteria (or vice versa). Disagreements between reviewers were resolved by consensus, with a third reviewer (MAH) available for arbitration. Most studies defined a clear purpose of assessing the validity and/or reliability of a given IMU outcome in healthy adults, however a number of studies addressed more advanced problems (e.g., clinical populations or new techniques) but still presented results that met our criteria.

### Methodological quality

Study quality was assessed by two independent reviewers (JFE and AG) using a modified version of the Critical Appraisal of Study Design for Psychometric Articles [14], which we adapted to studies evaluating the psychometric properties of wearable sensors (Additional file 3). This modified evaluation form contains 12 items evaluating study quality in 5 categories: study question, study design, measurements, analyses, and recommendations. Each item is scored as 2 (satisfactory), 1 (partially satisfactory), or 0 (unsatisfactory), with a total possible score out of 24 converted to a percentage. Raters were blinded to any identifiable information (e.g., author names, study title, publication year, journal) to avoid bias in their quality assessment. Initially, both raters evaluated two articles, after which they met to discuss each item to clarify their meaning and interpretation. The same process was repeated for each subsequent block of 20 articles. An intraclass correlation coefficient [ICC (3,1)] was calculated to evaluate pre-consensus inter-rater reliability of the total score. Disagreements were discussed and resolved through face-to-face meetings. If a consensus could not be reached, a third rater (DK) served as the tiebreaker. Studies obtaining a quality score between 85 and 100% were classified as high quality (HQ), those scoring between 70 and 85% were classified as moderate quality (MQ) and studies obtaining between 50 and 70% were classified as low quality (LQ). Studies rating below 50% were considered very low quality (VLQ) and were excluded from the quantitative synthesis. However, all studies were still included in the qualitative synthesis. Quality assessment scoring was then used to determine the strength of recommendations [15].

### Data extraction

Data were extracted from the included studies by one reviewer (NMK) and checked for accuracy by a second (JMC). Extracted data consisted of study design, sample demographics, inertial sensor specifications and placements, as well as each biomechanical outcome of interest and their reported statistical outcomes. While all statistical outcomes were extracted for the qualitative assessments, data pooling was a priori set to assess only the Pearson correlation coefficients (r) and ICCs for validity and reliability, respectively.

### Data pooling

Data pooling was facilitated with a multistage grouping of outcomes. First, all extracted outcomes were dichotomized as assessing either validity or reliability. Outcomes were then separated into overarching outcome groups (e.g., spatiotemporal, kinematic, other), before being grouped by specific outcome names (e.g., step time, stride time, step length, etc.) and finally sensor locations (e.g., foot, shank, thigh, back, etc.). For example, all assessments of “step time” would be grouped together, but further separated based on the placement of the inertial sensor. Data were not further pooled by type of sensor (e.g., accelerometer vs. gyroscope) or algorithm used. Therefore, a single study may contribute to multiple independent data poolings based on validity or reliability, outcome measure, and sensor placements. Biomechanical outcomes with three or more independent study samples using the same sensor location and reporting the desired statistical outcomes (i.e., r, ICC) were quantitatively synthesized. Agreement metrics (i.e., ICC and r) were interpreted as poor (< 0.500), moderate (0.500–0.749), good (0.750–0.899), and excellent (≥0.900).

Data for validity and reliability outcomes were meta-analyzed based on the r and ICC, respectively, and 95% confidence intervals were generated using a random-effects model (R version 3.6.0 using the meta package with the metacor function [16]). Weighting of individual point estimates was based on study sample sizes. Given the non-normality of Pearson correlation coefficients and ICCs, point estimates were variance-stabilized using Fisher’s z-transform [17]. In all cases where an ICC was reported, and as far as we could determine given the information available, the number of measures or comparators was m = 2; therefore, Fisher’s z-transform applied similarly to both r and ICC. However, for ICCs the standard error was adjusted to 1/√(N-3/2) following previous recommendations [18]. Data were then transformed back to their respective original outcome measures for reporting. Heterogeneity was examined using τ^2^, I^2^ and Cochran’s Q statistic where τ^2^ = 0 suggests no heterogeneity, I^2^ values < 25, 26–50%, and > 75% suggest low, moderate and high heterogeneity [19], and a significant Q statistic indicated that the studies do not share similar effects. Results of the meta-analysis were interpreted using the same agreement metric definitions as outlined above.

Alternatively, qualitative interpretation was conducted on outcomes that were unable to be quantitatively pooled. Additional error metrics (i.e., root-mean-square error (RMSE), standard error of measurement (SEM), minimum detectable change (MDC), limits of agreement (LoA)) were included in this qualitative synthesis to support our interpretations [15]:

- Strong evidence: multiple HQ or MQ studies with consistent results.
- Moderate evidence: multiple studies, including at least one HQ study or multiple MQ studies, presenting consistent results.
- Limited evidence: multiple LQ studies with inconsistent results, or one HQ/MQ study.
- Conflicting evidence: multiple studies providing inconsistent results, regardless of the methodological quality.
- Very limited evidence: only one LQ or MQ study or multiple VLQ

## Results

### Search results

**Fig. 1:**
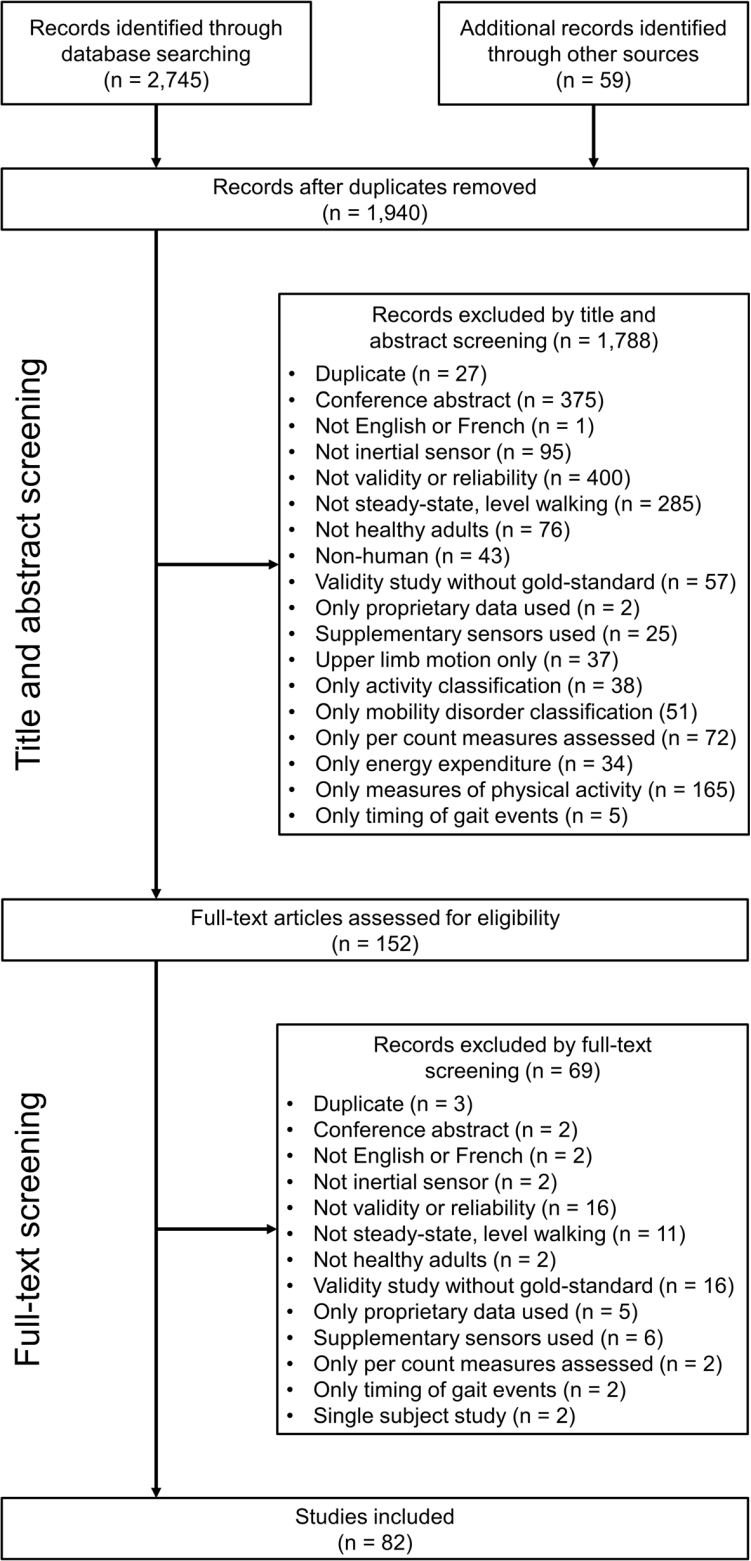
Flowchart of the systematic review selection process

**Fig. 2:**
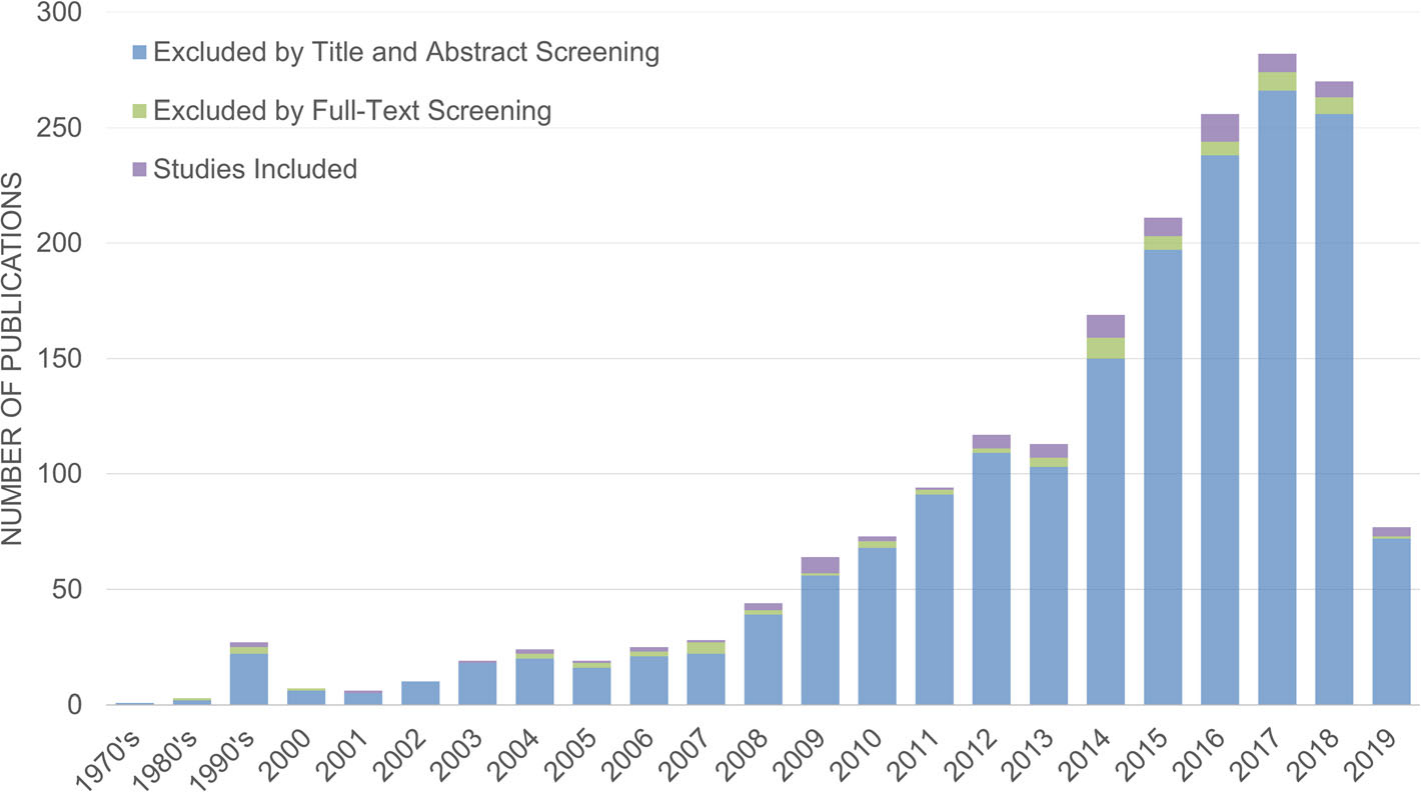
Number of studies identified, excluded, and included by years

Our search strategy identified a total of 2804 articles. Following the removal of duplicates, screening of titles/abstracts, and full-text screening, 82 articles [20–101] were included in the current review (Fig. 1). We did not set a date range on the search; however, the number of papers in this area was found to increase heavily from 2008 to 2014, with > 50% of the included papers published within approximately 5 years, and > 85% within 10 years (Fig. 2).

### Methodological quality

Only 1 article was rated as HQ, 13 as MQ, 50 as LQ and 18 as VLQ (Table 1). Agreement between both raters reached a single-measures ICC (3,1) of 0.83 [95% C.I. 0.75, 0.89). The items for which articles generally scored higher were “1- Background and research question” and “9- Organization and completeness of study results”. In contrast, 81 papers (95%) did not provide any justification about their sample size and/or appeared to be underpowered.

**Table 1:** Quality assessment scoring of 82 included studies

Study Information			Study Ques.	Study Design					Measurement		Analyses			Rec	Total	
Author	Year	Ref	Q1	Q2	Q3	Q4	Q5	Q6	Q7	Q8	Q9	Q10	Q11	Q12	/24	%
Abhayasinghe	2019	[20]	1	0	1	2	0	N/A	0	0	2	1	0	0	7	31.8%*
Al-Amri	2018	[21]	2	1	1	2	2	2	2	2	2	1	1	2	20	83.3%
Allseits	2018	[22]	2	1	1	2	0	N/A	0	1	2	2	0	2	13	59.1%*
Allseits	2017	[23]	2	1	1	2	0	N/A	1	2	2	2	0	2	15	68.2%*
Aminian	2004	[24]	1	0	0	2	0	N/A	1	2	2	1	0	1	10	45.5%*
Atallah	2014	[25]	2	1	2	2	0	N/A	0	1	2	2	1	0	13	59.1%*
Backhouse	2013	[26]	2	1	1	2	0	N/A	1	1	2	2	0	1	13	59.1%*
Bautmans	2011	[27]	2	2	0	2	0	2	1	1	2	2	2	2	18	75.0%
Ben Mansour	2015	[28]	2	0	0	2	0	N/A	1	1	2	0	0	1	9	40.9%*
Benoussaad	2016	[29]	2	1	0	2	0	N/A	1	1	1	0	0	1	9	40.9%*
Bertoli	2018	[30]	2	1	0	2	1	N/A	2	2	2	2	0	2	16	72.7%*
Bolink	2016	[31]	2	1	1	2	0	N/A	2	1	2	2	1	2	16	72.7%*
Bruijn	2010	[32]	2	0	1	2	0	N/A	1	2	1	1	1	1	12	54.5%*
Buganè	2012	[33]	2	1	1	2	0	N/A	1	1	2	0	0	1	11	50.0%*
Byun	2016	[34]	2	2	1	2	0	N/A	1	2	2	1	2	1	16	72.7%*
Chalmers	2014	[35]	2	0	0	2	0	N/A	1	0	1	0	0	0	6	27.3%*
Chapman	2019	[36]	1	0	0	2	0	N/A	0	2	2	2	0	1	10	45.5%*
Charlton	2019	[37]	2	1	2	2	2	2	2	0	2	2	2	2	21	87.5%
Cole	2014	[38]	2	1	1	2	0	N/A	1	1	2	2	1	2	15	68.2%*
Cooper	2009	[39]	2	1	0	2	0	N/A	0	2	1	0	0	0	8	36.4%*
Dalton	2013	[40]	2	1	0	2	0	N/A	1	1	2	1	1	2	13	59.1%*
Del Din	2016	[41]	2	1	1	2	0	N/A	1	2	2	1	1	2	15	68.2%*
Esser	2009	[42]	0	0	1	2	0	N/A	1	0	1	1	1	1	8	36.4%*
Furrer	2015	[43]	2	1	1	2	0	N/A	2	1	2	2	1	1	15	68.2%*
Godfrey	2015	[44]	2	1	1	2	0	N/A	1	1	2	1	1	2	14	63.6%*
Gonzalez	2016	[45]	2	1	1	2	1	N/A	1	1	2	0	0	1	12	54.5%*
Gorelick	2009	[46]	2	1	1	2	0	2	1	2	2	1	2	2	18	75.0%
Greene	2012	[47]	2	1	2	2	0	N/A	2	2	2	1	0	0	14	63.6%*
Greene	2010	[48]	2	0	1	2	0	N/A	2	2	2	1	0	1	13	59.1%*
Hamacher	2014	[49]	1	0	1	1	0	2	1	0	2	2	1	1	12	50.0%
Hamacher	2015	[50]	2	0	1	1	0	2	0	0	2	2	2	2	14	58.3%
Hartmann	2009	[51]	2	2	1	2	0	N/A	1	2	2	2	1	2	17	77.3%*
Hartmann	2009	[52]	2	2	1	2	0	2	1	1	2	2	1	2	18	75.0%
Henriksen	2004	[53]	2	1	1	1	0	2	0	2	2	1	2	2	16	66.7%
Huang	2016	[54]	2	1	1	2	0	N/A	1	2	2	0	0	2	13	59.1%*
Hundza	2014	[55]	2	0	0	2	0	N/A	1	0	2	0	0	2	9	40.9%*
Jarchi	2014	[56]	2	0	1	2	0	N/A	1	1	2	2	1	2	14	63.6%*
Karatsidis	2019	[57]	2	1	1	2	0	N/A	2	0	2	1	1	2	14	63.6%*
Kavanagh	2006	[58]	2	1	2	2	0	2	2	2	2	1	1	2	19	79.2%
Kitagawa	2016	[59]	2	1	0	1	0	N/A	0	0	1	0	0	2	7	31.8%*
Kluge	2017	[60]	2	1	1	2	0	0	1	1	2	1	1	1	13	54.2%
Köse	2012	[61]	2	0	1	1	0	N/A	0	0	2	0	0	1	7	31.8%*
Lebel	2017	[62]	2	1	0	2	0	N/A	1	1	2	1	1	2	13	59.1%*
L’Hermette	2008	[63]	1	1	0	1	0	N/A	1	0	1	1	0	1	7	31.8%*
Liikavainio	2007	[64]	2	1	1	1	0	2	2	2	2	0	1	2	16	66.7%
Liu	2009	[65]	2	1	0	1	0	N/A	0	1	2	1	0	2	10	45.5%*
Lord	2008	[66]	0	0	1	2	0	N/A	0	0	2	1	2	1	9	40.9%*
Lyytinen	2016	[67]	2	1	0	2	0	2	1	2	2	1	1	2	16	66.7%
Maffiuletti	2008	[68]	2	1	1	2	0	N/A	2	1	2	1	0	2	14	63.6%*
Manor	2018	[69]	2	2	1	2	0	2	0	0	1	1	1	2	14	58.3%
Mariani	2012	[70]	2	1	1	2	0	N/A	1	1	2	2	0	2	14	63.6%*
Mariani	2013	[71]	2	1	1	2	0	N/A	1	1	2	1	0	2	13	59.1%*
McGrath	2012	[72]	2	0	1	2	0	N/A	1	2	2	1	1	1	13	59.1%*
Moe-Nilssen	1998	[73]	2	0	0	1	0	2	1	1	2	2	1	2	14	58.3%
Nishiguchi	2012	[74]	2	1	0	0	0	2	1	1	2	1	1	1	12	50.0%
Ohtako	2001	[75]	2	0	0	2	0	N/A	0	1	1	1	0	2	9	40.9%*
Orlowski	2017	[76]	2	1	1	2	0	2	1	1	2	2	2	2	18	75.0%
Pepa	2017	[77]	2	0	1	2	0	N/A	0	2	2	2	0	1	12	54.5%*
Reynard	2014	[78]	2	2	1	1	0	2	1	0	2	2	2	2	17	70.8%
Sabatini	2015	[79]	2	1	1	2	0	N/A	2	2	1	2	0	2	15	68.2%*
Saremi	2006	[80]	2	0	1	1	0	2	2	1	2	0	0	2	13	54.2%
Schmitz-Hübsch	2016	[81]	2	1	1	2	0	N/A	0	1	2	2	0	1	12	54.5%*
Sejdic	2015	[82]	2	0	1	2	0	N/A	1	2	2	0	0	2	12	54.5%*
Selles	2005	[83]	2	1	0	2	0	N/A	1	2	2	1	1	1	13	59.1%*
Senden	2009	[84]	2	0	1	1	0	2	1	1	2	1	1	1	13	54.2%
Sijobert	2015	[85]	2	1	1	2	0	N/A	1	2	2	0	0	0	11	50.0%*
Silsupadol	2017	[86]	2	1	1	2	0	N/A	1	2	2	2	1	2	16	72.7%*
Steins	2014	[87]	1	0	1	1	0	N/A	1	0	2	2	2	1	11	50.0%*
Storm	2016	[88]	2	0	1	2	0	N/A	1	2	2	0	0	2	12	54.5%*
Teufl	2019	[89]	2	0	0	1	0	2	2	1	2	1	1	2	14	58.3%
Teufl	2018	[90]	2	1	1	1	0	2	2	0	2	1	2	2	16	66.7%
Trojaniello	2014	[91]	2	1	1	2	0	N/A	1	0	2	0	0	1	10	45.5%*
Trojaniello	2014	[92]	2	1	1	2	0	N/A	2	2	1	2	0	2	15	68.2%*
Trojaniello	2015	[93]	2	1	1	2	0	N/A	2	2	2	0	0	2	14	63.6%*
van der Straaten	2018	[94]	2	2	1	2	0	2	1	0	2	2	2	2	18	75.0%
van Schooten	2013	[95]	2	0	1	2	0	2	1	0	2	2	1	2	15	62.5%
Washabaugh	2017	[96]	1	1	1	2	0	1	0	1	2	2	1	2	14	58.3%
Wundersitz	2015	[97]	2	1	1	2	0	N/A	0	2	2	2	1	1	14	63.6%*
Xia	2017	[98]	2	1	0	2	0	N/A	2	1	2	1	1	1	13	59.1%*
Zhang	2013	[99]	1	1	1	2	0	N/A	2	0	1	0	1	0	9	40.9%*
Zijlstra	2013	[100]	2	1	1	1	0	2	1	1	2	1	1	2	15	62.5%
Zijlstra	2003	[101]	2	1	0	1	0	N/A	0	1	1	0	0	1	7	31.8%*

*Percentage calculated out of 22 as studies did not qualify for reliability and were not assessed on Q6 for sample retention

### Study characteristics

The 82 studies included in this review assessed biomechanical outcomes in walking using a variety of IMUs. The most common IMU system used was Xsens Technologies (*n* = 9), followed by Opal (*n* = 7), and finally Dynaport (*n* = 5) and Shimmer (*n* = 5). The most common sampling frequency used to assess walking was 100 Hz (range: 25-2000 Hz). Lastly, data from 1510 healthy adults were included across these studies (mean (sd) sample size: 18 (17) participants; median sample size: 12 participants; range: 2–95 participants). See Table 2 and Table 3 for breakdown of study characteristics separated based on validity and reliability, respectively.

**Table 2:** Details of studies assessing validity for spatiotemporal (ST), kinematic (KIN), and other biomechanical outcomes (OTHER)

Author	Year	Ref	n	Age	ST	KIN	OTHER	Sensor	Hz	Gold Standard System
Abhayasinghe	2019	[20]	19	*		X		MPU-9150 (InvenSense)	100	Motion Capture Camera System (Vicon Motion Systems, Oxford, UK)
Al-Amri	2018	[21]	25	35		X		Xsens MTw (Xsens Technologies BV, Netherlands)	60	Motion Capture Camera System (Vicon Motion Systems, Oxford, UK)
Allseits	2017	[23]	11	32	X			3D gyroscope (no manufacturer listed)	50	Instrumented Walkway (Matscan, Tekscan, Inc., Boston, MA)
Allseits	2018	[22]	11	32	X			3D gyroscope (no manufacturer listed)	*	Instrumented Walkway (Matscan, Tekscan, Inc., Boston, MA)
Aminian	2004	[24]	9	63	X	X		Physilog (BioAGM, CH)	200	Motion Capture Camera System and Force Plates (ELITE System, BTS, Milan, Italy)
Atallah	2014	[25]	14	40	X			3D accelerometer (no manufacturer listed)	130	Instrumented Treadmill (h/p/cosmos, Munich, Germany)
Backhouse	2013	[26]	12	42	X			IDEEA (MiniSun LLC., Fresno, CA)	*	Instrumented Walkway (GAITRite, CIR Systems Inc., Franklin, NJ)
Ben Mansour	2015	[28]	10	29	X			MMA8453Q (Freescale Semiconductor); L3G4200D (STMicroelectronics)	200	Instrumented Treadmill (ADAL 3D, Medical Development, Tecmachine Hef, France)
Benoussaad	2016	[29]	10	27	X			HikoB Fox (HikoB Villeurbanne, France)	200	Motion Capture Camera System (Vicon Motion Systems, Oxford, UK)
Bertoli	2018	[30]	80	74	X			Opal (Mobility Lab, APDM Inc., Portland, OR)	128	Instrumented Walkway (Zeno Walkway, Prokinetics LLC., Havertown, PA)
Bolink	2016	[31]	17	26		X		Microstrain Inertia-Link	100	Motion Capture Camera System (Vicon Motion Systems, Oxford, UK)
Bruijn	2010	[32]	9	*			X	PI-node (Philips, The Netherlands)	50	Motion Capture Camera System (Optotrak 3020, NDI, Waterloo, ON)
Buganè	2012	[33]	22	24/27	X			Free4Act (F4A)	50	Motion Capture Camera System (Vicon Motion Systems, Oxford, UK)
Byun	2016	[34]	82	69	X			FITMETER (FitLifeInc, Suwon, Korea)	32	Instrumented Walkway (GAITRite, CIR Systems Inc., Franklin, NJ)
Chalmers	2014	[35]	11	22		X		ADXL345 (Analog Devices); LPR450 (STMicroelectronics)	60	Motion Capture Camera System (No Brand Reported)
Chapman	2019	[36]	2	50		X		APDM	128	Motion Capture Camera System (OptiTrack, Natural Point, Inc., Corvallis, OR)
Cole	2014	[38]	24	71/23			X	Inertial Cube3 (InterSense Inc., Bedford, MA, USA)	100	Motion Capture Camera System (Vicon Motion Systems, Oxford, UK)
Cooper	2009	[39]	7	30		X		IMU (no manufacture/model reported)	100	Motion Capture Camera System (Qualisys, Sweden)
Dalton	2013	[40]	10	57	X		X	AD_BRC	250	Instrumented Walkway (GAITRite, CIR Systems Inc., Franklin, NJ)
Del Din	2016	[41]	30	67	X			Axivity AX3, UK	50	Instrumented Walkway (GAITRite, CIR Systems Inc., Franklin, NJ)
Esser	2009	[42]	5	23			X	Xsens MTx (Xsens Technologies BV, Netherlands)	100	Motion Capture Camera System (Qualisys, Göteborg, Sweden)
Furrer	2015	[43]	22	27			X	Smartphone: Desire HD, HTC Corp, Taiwan	50	Motion Capture Camera System (Vicon Motion Systems, Oxford, UK); Force Plates (OR 6, AMTI, Watertown, MA)
Godfrey	2015	[44]	40Y/37O	29/64	X			Axivity AX3, UK	100	Instrumented Walkway (GAITRite, CIR Systems Inc., Franklin, NJ)
Gonzalez	2016	[45]	5Y/5O	24/68	X			LPMS-B (LP Research, Japan)	*	Instrumented Walkway (GAITRite, CIR Systems Inc., Franklin, NJ)
Greene	2012	[47]	7	*	X			Shimmer (Shimmer Sensing, Dublin, IR)	102	Instrumented Walkway (GAITRite, CIR Systems Inc., Franklin, NJ)
Greene	2010	[48]	9	30	X			Shimmer (Shimmer Sensing, Dublin, IR)	102	Motion Capture Camera System (CODA Motion Analysis Leicestershire, UK)
Hartmann	2009	[51]	23	77	X			DynaPort (McRoberts BV, Hague, Netherlands)	100	Instrumented Walkway (GAITRite, CIR Systems Inc., Franklin, NJ)
Huang	2016	[54]	13	30		X		Invensense MPU6050 (San Jose, CA)	100	Motion Capture Camera System (Vicon Motion Systems, Oxford, UK)
Hundza	2014	[55]	7	30	X			Microelectromechanical systems (no manufacture listed)	40	Instrumented Walkway (GAITRite, CIR Systems Inc., Franklin, NJ)
Jarchi	2014	[56]	10	*	X			E-AR Sensor (Sensixa Ltd., London, UK)	130	Instrumented Treadmill (Gaitway, Kistler Instrument Corp, Amherst, US)
Karatsidis	2019	[57]	11	31		X		Xsens MVm (Xsens Technologies BV, Netherlands)	240	Motion Capture Camera System (Qualisys, Göteborg, Sweden); Force Plates (OR 6, AMTI, Watertown, MA)
Kitagawa	2016	[59]	10	23	X			TSND121 (ATR-Promotions, Japan)	200	Motion Capture Camera System (Motion Analysis Corp, Rohnert Park, CA)
Köse	2012	[61]	9	31	X			Sensorize (FreeSense ApS, Denmark)	100	Motion Capture Camera System (BTS, Milan, Italy)
Lebel	2017	[62]	20	50		X		IGS-180 (Synertial; Inertial Labs)	60	Motion Capture Camera System (Vicon Motion Systems, Oxford, UK)
L’Hermette	2008	[63]	15	23	X		X	ADXL105-EM3 (Analog Devices)	100	Motion Capture Camera System (Vicon Motion Systems, Oxford, UK)
Liu	2009	[65]	8	25			X	MM-2860 (Sunhayato, Japan)	*	Motion Capture Camera System (NAC Image Technology, Tokyo, Japan)
Lord	2008	[66]	11	73	X			Vitaport Activity Monitor (TEMEC Instruments Inc., Netherlands)	25	Instrumented Walkway (GAITRite, CIR Systems Inc., Franklin, NJ)
Maffiuletti	2008	[68]	10	34	X			IDEEA (MiniSun LLC, Fresno, CA)	32	Force Plates (Kistler Instrumente AG, Winterthur, Switzerland)
Manor	2018	[69]	14	30	X			iPhone 4 s (Apple, CA)	100	Instrumented Walkway (GAITRite, CIR Systems Inc., Franklin, NJ)
Mariani	2012	[70]	12	32	X			Physilog (BioAGM, CH)	200	Motion Capture Camera System (Vicon Motion Systems, Oxford, UK)
Mariani	2013	[71]	10	*	X			Physilog (BioAGM, CH)	200	Pressure Insoles (Pedar-X, Novel, DE)
McGrath	2012	[72]	5	*	X			Shimmer (Shimmer Sensing, Dublin, IR)	102	Motion Capture Camera System (CODA Motion Analysis, Leicestershire, UK)
Ohtako	2001	[75]	6	*		X		ADXL05 (Analog Devices); ENC03J (Murata)	100	Motion Capture Camera System (Vicon Motion Systems, Oxford, UK); Force Plates (OR 6, AMTI, Watertown, MA)
Pepa	2017	[77]	11	*	X			iPhone 4 s (Apple, CA)	100	Motion Capture Camera System (ELITE System, BTS, Milan, Italy)
Sabatini	2015	[79]	9	30			X	BMA180 (Bosch); ITG-3200 (InvenSense); HMC5843 (Honeywell)	100	Motion Capture Camera System (Vicon Motion Systems, Oxford, UK)
Saremi	2006	[80]	8	*	X			IDEEA (MiniSun LLC, Fresno, CA)	32	Pressure Insoles (B&L Engineering, Los Angeles, CA)
Schmitz-Hübsch	2016	[81]	9	50	X			Opal (Mobility Lab, APDM Inc., Portland, OR)	128	Instrumented Walkway (GAITRite, CIR Systems Inc., Franklin, NJ)
Sejdic	2015	[82]	14	74	X			MMA7260Q (Freescale Semiconductor)	100	Motion Capture Camera System (OptiTrack, Natural Point, Inc., Corvallis, OR)
Selles	2005	[83]	10	29	X			ICSensors 3021 (ICSensors Inc., Fremont, CA)	500	Force Plates (Kistler Instrumente AG, Winterthur, Switzerland)
Sijobert	2015	[85]	10	*	X			HikoB Fox (HikoB Villeurbanne, France)	200	Instrumented Walkway (GAITRite, CIR Systems Inc., Franklin, NJ)
Silsupadol	2017	[86]	34	23/74	X			Vivo X5 (Android 4.4.4)	100	Instrumented Walkway (GAITRite, CIR Systems Inc., Franklin, NJ)
Steins	2014	[87]	10	26			X	iPod Touch 4th generation (Apple, CA)	100	Motion Capture Camera System (Qualisys, Sweden)
Storm	2016	[88]	10	28	X			Opal (Mobility Lab, APDM Inc., Portland, OR)	128	Pressure Insoles (F-Scan 3000E, Tekscan, Inc., Boston, MA)
Teufl	2019	[89]	24	*	X			Xsens MTw Awinda (Xsens Technologies BV, Netherlands)	60	Motion Capture Camera System (OptiTrack, Natural Point, Inc., Corvallis, OR)
Teufl	2018	[90]	28	24		X		Xsens MTw Awinda (Xsens Technologies BV, Netherlands)	60	Motion Capture Camera System (OptiTrack, Natural Point, Inc., Corvallis, OR)
Trojaniello	2014	[91]	14	32	X			Opal (Mobility Lab, APDM Inc., Portland, OR)	128	Motion Capture Camera System (Vicon Motion Systems, Oxford, UK); Force Plates (OR 6, AMTI, Watertown, MA)
Trojaniello	2014	[92]	10	70	X			Opal (Mobility Lab, APDM Inc., Portland, OR)	128	Instrumented Walkway (GAITRite, CIR Systems Inc., Franklin, NJ)
Trojaniello	2015	[93]	10	70	X			Opal (Mobility Lab, APDM Inc., Portland, OR)	128	Instrumented Walkway (GAITRite, CIR Systems Inc., Franklin, NJ)
Washabaugh	2017	[96]	11	24	X			Opal (Mobility Lab, APDM Inc., Portland, OR)	*	Instrumented Treadmill (Bertec, Columbus, OH)
Wundersitz	2015	[97]	39	24			X	Minimax S4 (Catapult Innovations, Australia)	100	Motion Capture Camera System (Motion Analysis Corp, Rohnert Park, CA)
Xia	2017	[98]	14	25		X		MPU9150 (Invensense, USA)	100	Motion Capture Camera System (Vicon Motion Systems, Oxford, UK)
Zhang	2013	[99]	10	24		X		Xsens MVm (Xsens Technologies BV, Netherlands)	100	Motion Capture Camera System (Optotrak 3020, NDI, Waterloo, ON)
Zijlstra	2003	[101]	15	23	X			Kistler accelerometer	100	Instrumented Treadmill (No Brand Reported)

*Information not reported

**Table 3:** Details of studies assessing reliability for spatiotemporal (ST), kinematic (KIN), and other biomechanical outcomes (OTHER)

Author	Year	Ref	n	Age	ST	KIN	OTHER	Sensor	Hz
Al-Amri	2018	[21]	24	35		X		Xsens MTw Awinda (Xsens Technologies BV, Netherlands)	60
Bautmans	2011	[27]	20O/20Y	79/22	X		X	DynaPort (McRoberts BV, The Hague, The Netherlands)	100
Charlton	2019	[37]	20	28.3		X		Invensense MPU6050 (San Jose, CA)	100
Gorelick	2009	[46]	8F/10M	25/31	X			IDEEA (MiniSun LLC., Fresno, CA)	*
Hamacher	2014	[49]	19	71	X			Xsens MTw (Xsens Technologies BV, Netherlands)	75
Hamacher	2015	[50]	17O/12Y	71/26			X	Xsens MTw (Xsens Technologies BV, Netherlands)	75
Hartmann	2009	[52]	23	73	X			DynaPort (McRoberts BV, The Hague, The Netherlands)	100
Henriksen	2004	[53]	20	35	X		X	Meac-x (Mega Electronics Ltd., Kuopio, Finland)	250
Kavanagh	2006	[58]	8	23		X		ADXL202 (Analog Devices)	250
Kluge	2017	[60]	11	34	X			Shimmer (Shimmer Sensing, Dublin, IR)	102
Liikavainio	2007	[64]	10	29	X		X	Meac-x (Mega Electronics Ltd., Kuopio, Finland)	2000
Lyytinen	2016	[67]	9	23			X	Meac-x (Mega Electronics Ltd., Kuopio, Finland)	1000
Manor	2018	[69]	14	30	X			iPhone 4 s (Apple, CA)	100
Moe-Nilssen	1998	[73]	19	23			X	Logger Technologi HB (Ostragardsvagen, Sweden)	128
Nishiguchi	2012	[74]	30	21			X	Sony Ericsson, Xperia SO-01B	32
Orlowski	2017	[76]	25	26	X			Shimmer (Shimmer Sensing, Dublin, IR)	102
Reynard	2014	[78]	95	44			X	Physilog (BioAGM, CH)	200
Saremi	2006	[80]	12	31	X			IDEEA (MiniSun LLC., Fresno CA)	32
Senden	2009	[84]	24	21–60			X	McRoberts BV (Hague, Netherlands)	100
Teufl	2019	[89]	24	*	X			Xsens MTw Awinda (Xsens Technologies BV, Netherlands)	60
Teufl	2018	[90]	28	24		X		Xsens MVN (Xsens Technologies BV, Netherlands)	60
van der Straaten	2018	[94]	20	63		X		Xsens MVN (Xsens Technologies BV, Netherlands)	*
van Schooten	2013	[95]	20	29			X	DynaPort (McRoberts BV, Hague, Netherlands)	100
Washabaugh	2017	[96]	19	24	X			Opal (Mobility Lab, APDM Inc., Portland, OR)	*
Zijlstra	2013	[100]	20	74	X			DynaPort (McRoberts BV, Hague, Netherlands)	100

*Information not reported

### Validity

**Fig. 3:**
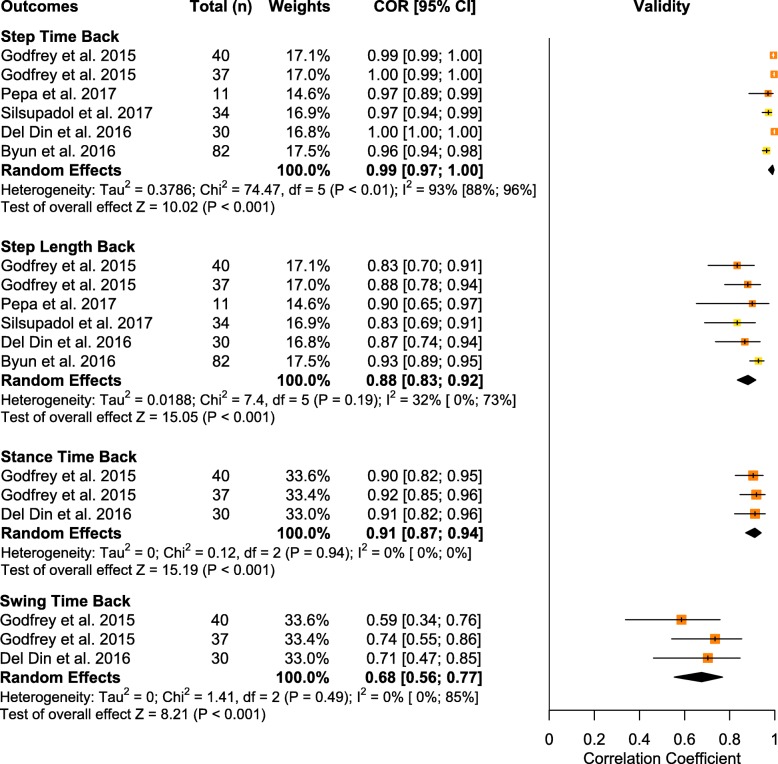
Forest plot of data pooling for spatiotemporal mean validity. Squares represent Pearson correlation coefficients and bars indicate 95% confidence intervals, with diamonds as pooled data. Methodological quality of each study is indicated by colour: HQ = green, MQ = yellow, LQ = orange, and VLQ = red

**Fig. 4:**
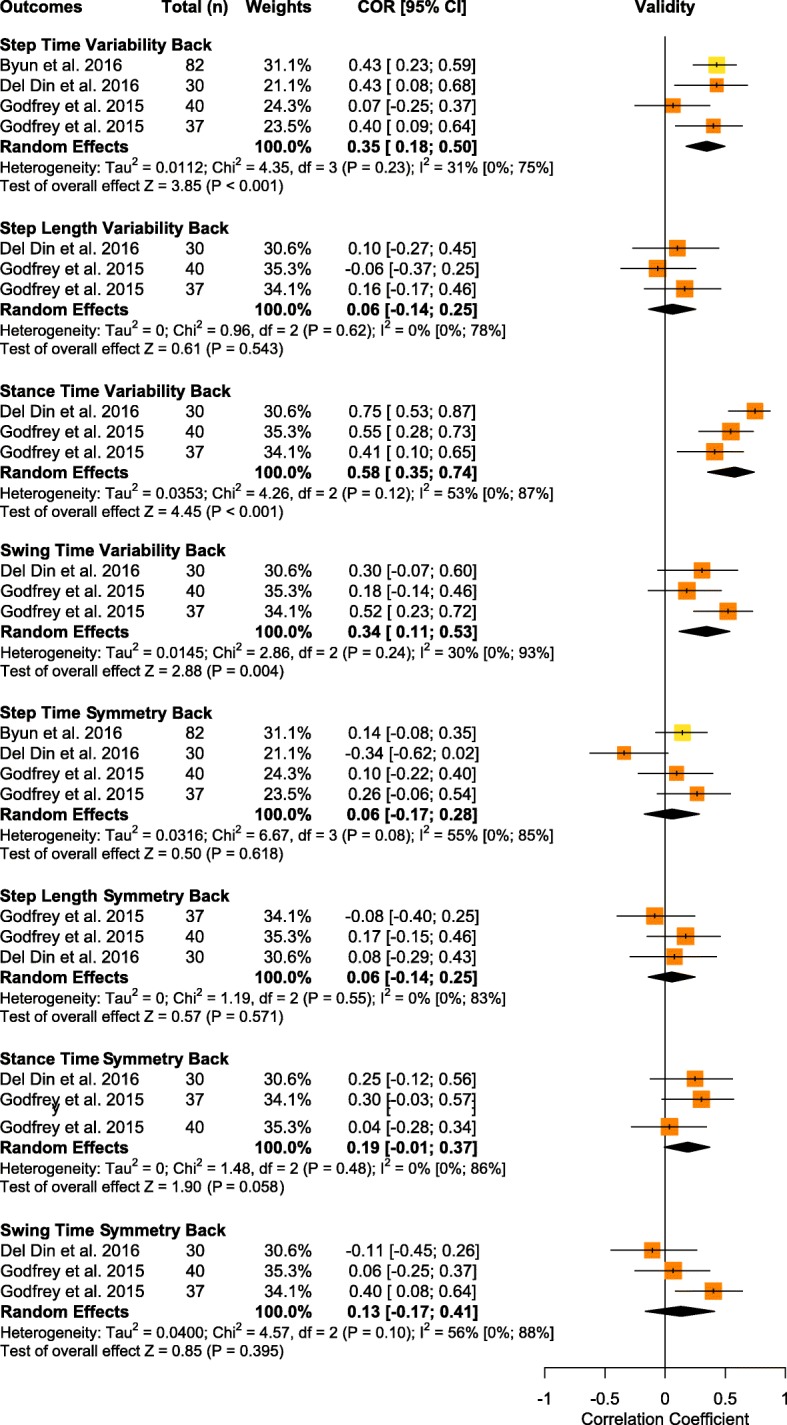
Forest plot of data pooling for spatiotemporal variability and symmetry validity. Squares represent Pearson correlation coefficients and bars indicate 95% confidence intervals, with diamonds as pooled data. Methodological quality of each study is indicated by colour: HQ = green, MQ = yellow, LQ = orange, and VLQ = red

Overall, a total of 23 spatiotemporal outcomes, 3D lower limb kinematics and kinetics, plus 7 other biomechanical outcomes were assessed across the 63 studies that examined IMU validity. From these outcomes, 12 spatiotemporal parameters presented sufficient study quality and statistical outcomes to allow for data pooling (Fig. 3 and Fig. 4). We were unable to meta-analyze kinematic/kinetic outcomes or other biomechanical outcomes, due to either a limited number of studies or, in many cases, a lack of consistency in data reporting, as many studies reported only RMSE or even a simple mean difference. Studies that were unable to be meta-analyzed were qualitatively summarized by outcomes and placements in Supplementary Table 1 for spatiotemporal outcomes, Supplementary Table 2 for kinematic/kinetic outcomes, and Supplementary Table 3 for other biomechanical outcomes. Therefore, the results presented in the following section represent only outcomes and placements which allowed for quantitative data pooling.

### Quantitative pooling of spatiotemporal outcomes for validity

#### Step time

Data from five low to moderate quality studies (contributing six independent study samples) suggests that the validity for step time measured with IMUs placed on the back was excellent (total *n* = 257; *r* = 0.99, 95% CI [0.97, 1.00], I^2^ = 93%, *p* < 0.001) [34, 41, 44, 77, 86]. An additional 10 studies that could not be pooled provided limited evidence for moderate to excellent validity of step times measured at the back or shank/ankle [28, 51, 61, 88, 91, 93].

#### Step length

Data from five low to moderate quality studies (contributing six independent study samples) suggests that the validity for step length measured with IMUs placed on the back was good (total *n* = 234; *r* = 0.88, 95% CI [0.83, 0.92]; I^2^ = 32%; *p* < 0.001) [34, 41, 44, 77, 86]. An additional study that could not be pooled provided limited evidence for excellent validity of step length measured at the back [51].

#### Stance time

Data from two low quality studies (contributing three independent study samples) suggests that the validity for stance time measured with IMUs placed on the back was excellent (total *n* = 107; *r* = 0.91, 95% CI [0.87, 0.94]; I^2^ = 0%; *p* < 0.001) [41, 44]. An additional 5 studies that could not be pooled provided limited evidence for moderate validity of stance times measured at the back [28, 82, 88, 91, 93].

#### Swing time

Data from two low quality studies (contributing three independent study samples) suggests that the validity of swing time measured with IMUs placed on the back was moderate (total *n* = 107, *r* = 0.68, 95% CI [0.56, 0.77]; I^2^ = 0%; *p* < 0.001) [41, 44]. An additional 3 studies that could not be pooled provided very limited evidence for moderate validity of swing times measured at the back [28, 91, 93].

#### Step time variability

Data from three low to moderate quality studies suggests that the validity of step time variability measured with IMUs placed on the back was poor (total *n* = 189, *r* = 0.35, 95% CI [0.18, 0.50]; I^2^ = 31%, *p* < 0.001) [34, 41, 44]. An additional 2 studies that could not be pooled provided limited evidence for excellent validity of step time variability measured at the back [51, 88].

#### Step length variability

Data from two low quality studies (contributing three independent study samples) suggests that the validity of step length variability measured with IMUs placed on the back was poor (total *n* = 107; *r* = 0.06, 95% CI [− 0.14, 0.25]; I^2^ = 0%, *p* = 543) [41, 44]. An additional study that could not be pooled provided limited evidence for poor validity of step length variability measured at the back [51].

#### Stance time variability

Data from two low quality two studies (contributing three independent study samples) suggests that the validity of stance time variability measured by IMUs placed at the back was moderate (total *n* = 107; *r* = 0.58, 95% CI [0.35, 0.74]; I^2^ = 0.53%; *p* < 0.001) [41, 44]. An additional study that could not be pooled provided very limited evidence for moderate validity of stance time variability measured at the back [88].

#### Swing time variability

Data from two low quality studies (contributing three independent study samples) suggests that the validity of swing time variability measured by IMUs placed at the back was poor (total *n* = 107; *r* = 0.34, 95% CI [0.11, 0.53]; I^2^ = 30%; *p* = 0.004) [41, 44].

#### Step time symmetry

Data from three low to moderate quality studies suggests that the validity of step time symmetry measured by IMUs placed at the back was poor (total *n* = 189; *r* = 0.06, 95% CI [− 0.17, 0.28]; I^2^ = 55%; *p* = 0.618) [34, 41, 44].

#### Step length symmetry

Data from two low quality studies (contributing three independent study samples) suggests that the validity of step length symmetry measured by IMUs placed at the back was poor (total *n* = 107; *r* = 0.06, 95% IC [− 0.14, 0.25]; I^2^ = 0%; *p* = 0.571) [41, 44].

#### Stance time symmetry

Data from two low quality studies (contributing three independent study samples) suggests that the validity of stance time symmetry measured by IMUs placed at the back was poor (total *n* = 107; *r* = 0.19, 95% CI [− 0.01, 0.37]; I^2^ = 0%; *p* = 0.058) [41, 44].

#### Swing time symmetry

Data from two low quality studies (contributing three independent study samples) suggests that the validity of swing time symmetry measured by IMUs placed at the back was poor (total *n* = 107; r = 0.13, 95% CI [− 0.17, 0.41]; I^2^ = 56%; *p* = 0.395) [41, 44].

### Reliability

**Fig. 5:**
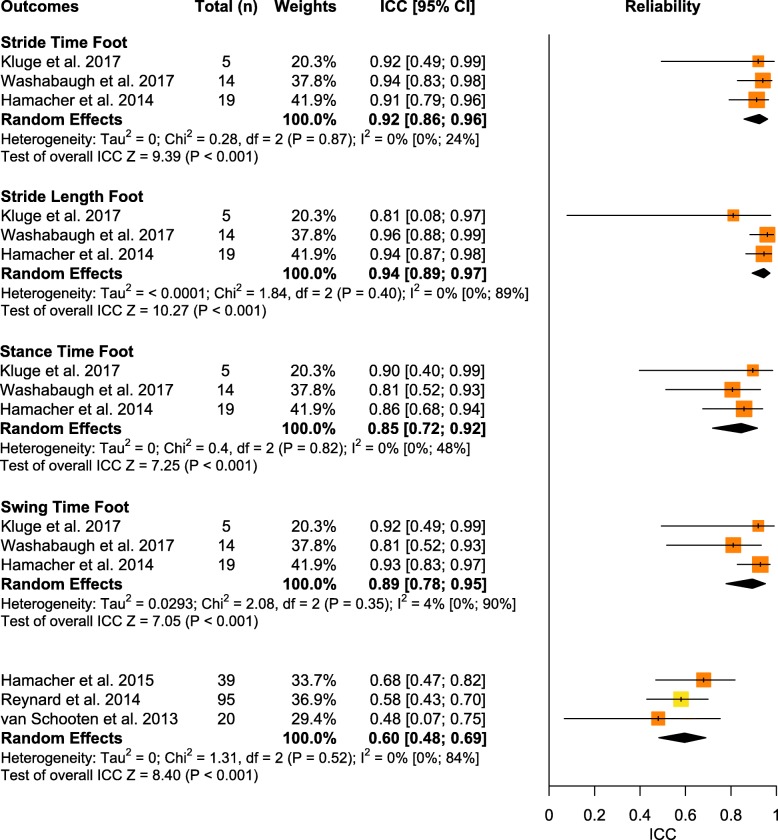
Forest plot of data pooling for spatiotemporal and other biomechanical outcome reliability. Squares represent intraclass correlation coefficients and bars indicate 95% confidence intervals, with diamonds as pooled data. Methodological quality of each study is indicated by colour: HQ = green, MQ = yellow, LQ = orange, and VLQ = red

Overall, a total of 15 spatiotemporal outcomes, 3D lower limb kinematics, and 8 other biomechanical outcomes were assessed across the 25 studies that examined IMU reliability (See Table 3). From this group, 4 spatiotemporal outcomes and 1 other biomechanical outcome presented sufficient study quality and statistical outcomes for meta-analysis (Fig. 5), but no kinematic outcomes were able to be pooled. Similar to validity, the inability to pool many outcomes was due to either a limited number of studies or, in many cases, a lack of consistency in data reporting. Studies that were unable to be pooled were qualitatively summarized by outcomes and placements in Supplementary Table 4 for spatiotemporal outcomes, Supplementary Table 5 for kinematic outcomes, and Supplementary Table 6 for other biomechanical outcomes.

### Quantitative pooling of spatiotemporal outcomes for reliability

#### Stride time

Data from three low quality studies suggests that the reliability of stride time measured by IMUs placed at the foot was excellent (total *n* = 38; ICC = 0.92, 95% CI [0.86, 0.96]; I^2^ = 0%; *p* < 0.001) [49, 60, 96].

#### Stride length

Data from three low quality studies suggests that the reliability of stride length measured by IMUs placed at the foot was excellent (total *n* = 38; ICC = 0.94, 95% CI [0.89, 0.97]; I^2^ = 0%; *p* < 0.001) [49, 60, 96].

#### Stance time

Data from three low quality studies suggests that the reliability of stance time measured by IMUs placed at the foot was good (total *n* = 38; ICC = 0.85, 95% CI [0.72, 0.92]; I^2^ = 0%, *p* < 0.001) [49, 60, 96].

#### Swing time

Data from three low quality studies suggests that the reliability of swing time measured by IMUs placed at the foot was good (total *n* = 38; ICC = 0.89, 95% CI [0.78, 0.95]; I^2^ = 4%; *p* < 0.001) [49, 60, 96].

### Quantitative pooling of other biomechanical outcomes for reliability

#### Local dynamic stability

Data from three low to moderate quality studies suggests that the reliability of a local dynamic stability outcome, namely short-term, maximum Lyapunov exponent in the mediolateral axis, measured by IMUs placed at the back was moderate (total *n* = 154; ICC = 0.60, 95% CI [0.48, 0.69]; I^2^ = 0%; *p* < 0.001) [50, 78, 95].

## Discussion

The aim of this review was to determine the validity and reliability of biomechanical outcomes derived from IMUs during healthy adult walking, with the hope that we could pool results to provide valuable recommendations based on this immense body of literature. While 82 studies, examining over 100 outcomes, were included in this review, we were able to conduct meta-analysis for only 17 outcomes. Moreover, most data pooling occurred from a limited number of studies (e.g., 3–5). Nevertheless, these findings were able to provide a much-needed synthesis of the validity and reliability data for spatiotemporal, kinematic/kinetic, and other biomechanical outcomes from IMUs, as well as important recommendations for future studies in this growing field of research.

Spatiotemporal parameters presented the most fertile ground to pool results and make recommendations. Most notably, step time and stride time presented the strongest body of evidence for excellent validity and reliability. Although pooling was only possible for step time validity (back) and stride time reliability (foot), the qualitative pooling of results across the back, foot, and other placements also provide relatively consistent, but limited, evidence (based on study quality) for excellent validity and reliability. This limited, but generally consistent evidence was similarly found for good to excellent validity and reliability of step length and stride length across a variety of placements (e.g., back, shank, foot). Lastly, stance time and swing time were examined in fewer studies but were still found to present good to excellent validity and reliability in all pooled data, except swing time validity (moderate validity). Qualitative pooling of these spatiotemporal parameters across a variety of placements generally supported this conclusion with good to excellent validity and reliability. Overall, these findings are supportive of the assessment of mean spatiotemporal outcomes using IMUs, but do not clearly identify any IMU placement to be superior to another. It was only the validity of mean stride length which demonstrated a potential advantage of an IMU at the foot (e.g., excellent validity) compared to the back (e.g., good validity), with reliability metrics remaining excellent at both placements. This provides evidence for improved results of length parameters measured at the foot compared to the back, as one might expect. However, there was only a single study assessing the validity of mean stride length at the back [51] and as such this should be interpreted with caution. To this point, many of the above recommendations were defined as “limited evidence”, but we would argue that this statement of “limited evidence” is primarily based on the limited quality of studies, rather than a limitation of the sensors and outcomes themselves.

Contrary to spatiotemporal mean outcomes, the validity and reliability of spatiotemporal variability and symmetry outcomes were less favourable. Specifically, the validity of pooled variability and symmetry outcomes (step time, step length, stance time, swing time) measured at the back were poor to moderate, with the qualitative pooling of results providing similar findings on a variety of variability outcomes and placements. The limited studies assessing reliability of these variability and symmetry outcomes fared slightly better, demonstrating poor to good reliability. In contrast to these findings, one study found excellent validity for step time variability [51]. Notably, this study also displayed the highest quality of any in this outcome category at 77.3%. Moreover, step time variability was calculated based from 4 separate walking trials, which may have improved their findings. Nevertheless, these results suggest that unlike mean spatiotemporal outcomes which may mask random error from step to step, variability measures (e.g., standard deviation of individual step or stride-based outcomes) are, by definition, more susceptible to these errors and also require strict and standardized protocols. In general, these findings are similar to a previous review of gait variability across a variety of measurement devices [102], further suggesting that it is more likely the protocol than the IMU itself that limits the validity and reliability of these variability measures. Further, while Lord et al. [102] provided some recommendations (e.g., minimum 12 steps, piloting reliability, etc.), there remains a need for better defined protocols and processing standards for spatiotemporal variability outcomes. For example, variability outcomes computed from, ideally, at least 30 continuous steps [103, 104], or to a lesser extent, multiple walking trials to reach this number [51, 105], may serve to improve the validity and reliability of these important outcomes.

Similar to recent reviews examining the validity and reliability of IMU-derived lower limb joint kinematics [12, 13], we were unable to pool any of these results. This inability to pool data remained even though we had a more homogenous cohort of studies (i.e., healthy adults during walking). Nevertheless, this improved homogeneity did allow us to draw more consistent qualitative interpretations for IMUs in healthy adult walking. For example, while our results support previous conclusions that IMUs provided better estimates of lower limb sagittal joint angles as compared to frontal or transverse angles [12, 13], we also found more consistent levels of good to excellent validity and reliability in the sagittal plane. Further, this translated to RMSEs (Supplementary Tables 2 and 5) approximately half that of previous reviews based on a variety of movements [12, 13]. Similarly, although frontal and transverse plane joint angles displayed less validity and reliability than sagittal joint angles, they were generally found to be moderate to excellent. While this supports the use of IMUs for the measurement of 3D lower limb joint angles, it should be noted that much of this evidence remains limited for the sagittal plane, and very limited for other planes. Therefore, future research should not only focus on improving these results by examining potential sources of error (e.g., orientation estimates, anatomical calibrations, soft-tissue artifacts, etc.), but doing so in more rigorous research designs. Lastly, in addition to joint angles, we found IMUs displayed excellent validity for obtaining segment angles at the foot, shank, and thigh. Although these findings are also drawn from very limited evidence, this more simplistic approach of measuring segment orientations does not lead to compounding levels of error from multiple sensors across a joint, and as such, may be a better use of IMUs if the information of interest can be derived from a single segment [62].

While IMUs offer the unique opportunity to collect a variety of other biomechanical outcomes, only the reliability results for measures of stability, regularity, and acceleration RMSE were found to have stronger than very limited evidence. Short-term local dynamic stability (mediolateral axis), assessing complex non-linear aspects of gait variability and control [78], was the only outcome to be meta-analyzed and demonstrated moderate reliability. Stride regularity and step symmetry outcomes, assessing the consistency of acceleration waveforms using an autocorrelation procedure [106], demonstrated good and moderate reliability, respectively, but only from qualitative pooling. Further, similar to measures of gait variability, there remains limited information on the best practices for collecting these data. Lastly, acceleration RMS outcomes reported by five studies demonstrated limited evidence for good to excellent reliability in individual axes but could not be meta-analyzed due to incompatibilities in statistical parameters. Together, these results are promising for the reliability of other biomechanical measures that track human motion, but require more high-quality studies to establish better standards for the reliability of these outcomes. While the lack of validity data on these biomechanical outcomes may also be limiting, the unique nature of these outcomes may make establishing a true gold standard validity to optical systems less necessary if more high-quality reliability evidence was present.

One of the most important findings from this review is the lack of high-quality evidence and appropriate statistical outcomes utilized in much of the research in this field. The methodological quality assessment was adapted to best rate IMU validity and reliability studies, and yet many scored poorly. Underpowered and/or unjustified sample sizes were the most glaring issue, with a lack of appropriate statistical outcomes being a common problem as well. For instance, many studies simply reported mean differences as a measure of validity or reliability, which only addresses the bias of the system and not the agreement. Alternatively, reporting only Pearson’s r does not describe any potential systematic bias between measures. Therefore, we strongly advocate for all future work in this area to not only include adequate and/or justified sample sizes [107], but more appropriate statistical outcomes. Specifically, we would advise future work to include both relative (e.g., r, ICC) and absolute (e.g., LOA, SEM) statistical metrics [108, 109]. Further, Bland and Altman plots provide an excellent method to visualize the distribution of scores, but they should always be accompanied with the bias (i.e., mean difference) and an estimate of precision (i.e., standard deviation or 95% confidence interval of mean difference), as well as the limits of agreement with an estimate of precision (95% confidence interval of limits of agreement [110];). While there may be additional metrics that can support the interpretation of results (e.g., RMSE, MDC, etc.), including the aforementioned relative and absolute statistical outcomes as a minimum will provide the reader with an excellent impression of the validity and/or reliability that can be expected on biomechanical outcomes derived from IMUs.

In addition to providing recommendations, we must also acknowledge the limitations in our study. First, we chose not to include per unit measures (counts, cadence, gait speed, etc.) as these can be determined based on post collection estimates (e.g., distance travelled over a given time period = gait speed) which would confound results. Similarly, we chose not to include the direct timing of gait events (e.g., initial contact, toe-off, etc.) as these define the precursors to spatiotemporal outcomes, but not the actual outcomes themselves. Also, due to the already large scope of this review, we did not include within-session reliability or between-session reliability where the device was not removed. For example, Moe-Nilssen [111] examined a variety of outcomes relevant to the current review, but data from that study were not included as the researchers did not remove the device between sessions, and was therefore assessing a different level of IMU reliability. Lastly, we attempted to separate outcomes by walking speed in our synthesis of data and whenever possible used normal or preferred speeds to best represent healthy adult gait. Nevertheless, there were several instances where this was not possible and, as such, some data has mixed speed results.

### Future directions

The findings from this comprehensive review and meta-analysis illustrate the vast and continually growing body of literature in this field. Nevertheless, even with this large body of literature, it remains difficult to synthesize findings due to a lack of study quality and standardized protocols. Therefore, we urge the IMU community to focus on quality over quantity in research, as more poor quality, limited sample size studies will not advance the field but only convolute the results. In addition to this general recommendation, we present four specific recommendations for future directions.

- IMUs consistently demonstrate at least moderate validity and reliability in assessing all mean spatiotemporal parameters. Further, excellent validity and reliability can be expected on measures of step and stride time and length measured at the back and lower limbs. Therefore, we do not recommend the need for future studies to address the validity and/or reliability of mean step and stride time and length during walking as a primary outcome.
- Measures of spatiotemporal parameter variability from IMUs demonstrate inconsistent levels of validity and reliability. However, these inconsistencies are more likely due to variable protocols (i.e., number of steps/trials) and processing techniques, rather than a flaw in the devices themselves. Therefore, future research should seek to identify optimal and standardized protocols and processing techniques best suited to assess measures of gait variability with IMUs.
- While joint kinematics generally demonstrate good to excellent validity and reliability in the frontal and sagittal plane, this information is often drawn from small studies with poor statistical measures. Future research in this area must improve study designs (e.g., justified sample sizes, appropriate statistical outcomes) in order to provide more high-quality evidence and recommendations on these important outcomes.
- Additional biomechanical outcomes such as a stability, regularity, and acceleration RMS demonstrate promising reliability. Unfortunately, much like gait variability, there is a lack information on optimal and standardized protocols. Moreover, similar to joint kinematics, there is a need for more high-quality study designs. Therefore, future research should seek to address the best practices for IMU measures such as stability, regularity, and acceleration RMS using appropriate sample sizes and statistical outcomes.

## Conclusion

The findings of this review demonstrate the excellent validity and reliability of IMUs for measuring mean step/stride time and length during walking, but caution the use of spatiotemporal variability and symmetry metrics without strict protocol. Further, this work tentatively supports the use of IMUs for joint angle measurement, especially in the sagittal plane, and other biomechanical outcomes such as stability, regularity, and segmental accelerations. Unfortunately, the strength of these recommendations are limited based on the paucity of high-quality studies for each outcome. Future work should seek to address these gaps by undertaking more rigorous study designs and statistical considerations for testing the validity and reliability of IMU-derived biomechanical outcomes in walking. We have provided several recommendations for future studies that will strengthen the quality of the results and provide better insights into the validity and reliability of IMUs for gait analysis.

## Supplementary information

**Additional file 1.** Complete Inclusion/Exclusion Criteria.

**Additional file 2.** Complete Search Strategy.

**Additional file 3.** Critical Appraisal of Study Design for Psychometric Articles.

**Additional file 4 Supplementary Table 1.** Qualitative summary of validity for spatiotemporal outcomes: r/ICC is presented as a weight average and range of reported values, while RMSE, Bias, and LOA widths are provided as the range of reported values. Gray shading identifies outcomes that have been quantitatively pooled in the results section. **Supplementary Table 2.** Qualitative summary of validity for other kinematic (and joint moment) outcomes: r/ICC is presented as a weight average and range of reported values, while RMSE, Bias, and LOA widths are provided as the range of reported values. **Supplementary Table 3.** Qualitative summary of validity for other biomechanical outcomes: r/ICC is presented as a weight average and range of reported values, while RMSE, Bias, and LOA widths are provided as the range of reported values. **Supplementary Table 4.** Qualitative summary of reliability for spatiotemporal outcomes: r/ICC is presented as a weight average and range of reported values, while SEM, MDC, Bias, and LOA widths are provided as the range of reported values. **Supplementary Table 5.** Qualitative summary of reliability for other kinematic outcomes: r/ICC is presented as a weight average and range of reported values, while SEM, MDC, Bias, and LOA widths are provided as the range of reported values. **Supplementary Table 6.** Qualitative summary of reliability for other biomechanical outcomes: r/ICC is presented as a weight average and range of reported values, while SEM, MDC, Bias, and LOA widths are provided as the range of reported values.

## Data Availability

Not applicable.

## Abbreviations

3D: Three Dimensional
IMU: Inertial Measurement Unit
ICC: Intraclass Correlation Coefficient
r: Pearson Correlation Coefficient
RMSE: Root-Mean-Square Error
SEM: Standard Error of Measurement
LoA: Limits of Agreement
HQ: High Quality
MQ: Moderate Quality
LQ: Low Quality
VLQ: Very Low Quality
