# Supplementary material for: Validity and reliability of wearable inertial sensors in healthy adult walking: a systematic review and meta-analysis
Source: J Neuroeng Rehabil. 2020 May 11;17:62. doi: 10.1186/s12984-020-00685-3 (PMC7216606; doi:10.1186/s12984-020-00685-3)
Supplement: Supplementary file 1 — Additional file 1. Complete Inclusion/Exclusion Criteria. [file 12984_2020_685_MOESM1_ESM.docx]

**Complete Inclusion/Exclusion Criteria**

For a study to be included in the review it must either:

1. report the **concurrent validity** (i.e., simultaneous collection) of biomechanical outcomes during **level overground or treadmill walking** as measured by inertial sensors* and compared to a gold standard**,
2. report the **test-retest reliability** (i.e., between-day, within-day, or between-tester; involving the same measure/device/placement with removal between session) of biomechanical outcomes during **level overground or treadmill walking** as measured by inertial sensors.

Additionally, studies must have included:

1. adults between 18+ years of age with no injury, pain, or musculoskeletal conditions that may affect their walking,
2. the analysis of inertial data/description of data analysis (i.e, not proprietary software/analysis),
3. at least one spatiotemporal, kinematic, or kinetic variable,
4. and be published in English or French.

**Definitions:**

****Inertial sensor*** was defined as a wearable sensor that utilizes any combination of accelerometer, gyroscope, and/or magnetometer signals.

*****Gold standard for spatiotemporal variables include***: Commercially available three-dimensional motion capture (i.e., stereophotogrammetry), force plate(s), instrumented mat (e.g., GAITRite), instrumented treadmill, or footswitch systems.

*****Gold standard for kinematic or kinetic variables***: Commercially available three-dimensional motion capture (i.e., stereophotogrammetry), force plate(s), and/or instrumented treadmill systems.

*Note: Custom devices or other inertial measurement systems would not qualify as gold standards.*

Studies were excluded from the review if they ONLY examined:

1. upper limb motion,
2. activity/movement classification,
3. per unit measurement (e.g., step count, cadence, velocity/speed)
4. gait events as outcomes (e.g., timing of initial contact)
5. energy expenditure,
6. measures of physical activity (i.e., counts),
7. non-human subjects, or
8. measures derived using supplementary sensors (e.g., global position system, footswitches, etc.).
