# Supplementary material for: Validity and reliability of wearable inertial sensors in healthy adult walking: a systematic review and meta-analysis
Source: J Neuroeng Rehabil. 2020 May 11;17:62. doi: 10.1186/s12984-020-00685-3 (PMC7216606; doi:10.1186/s12984-020-00685-3)
Supplement: Supplementary file 2 — Additional file 2. Complete Search Strategy. [file 12984_2020_685_MOESM2_ESM.docx]

**Complete Search Strategy**

Search strategy individually optimized for each database based on the four broad topics of inertial sensors, gait biomechanics, healthy adults, and validity/reliability.

**MEDLINE:**

**Inertial Sensors:** (wearable sensor* or inertial sensor* or inertial motion capture or inertial measurement unit* or imu or acceleromet* or gyroscop* or magnetomet* or smart phone*).mp. or exp accelerometry/

**Gait Biomechanics:** ((speed* or time* or length* or width or cadence* or spatiotemporal or analys* or kinematic* or kinetic* or biomechanic* or angle* or acceleration*).mp. adj5 (step or stride or gait* or walk* or ambulat* segment* or joint* or hip* or knee* or ankle* or torso* or center of mass or centre of mass or center of gravity or centre of gravity).mp.) or exp biomechanical phenomena/

**Healthy Adults:** ((Middle Aged/ or Aged/ or older.mp. or old* or young* or healthy or pain-free or able-bodied or asymptomatic or matched) adj5 (subject* or adult* or individual*).mp.) or exp control/ or exp control group/ or control*.mp.

**Validity/Reliability:** (valid* or reliab* or agreement*).mp. or exp "reproducibility of results"/

**EMBASE:**

**Inertial Sensors:** (wearable sensor* or smart phone* or inertial sensor* or inertial motion capture or inertial measurement unit* or imu or acceleromet* or gyrosco* or magnetomet*).mp. or exp accelerometer/

**Gait Biomechanics:** ((speed* or time* or length* or width or cadence* or spatiotemporal or analys* or kinematic* or kinetic* or biomechanic* or angle* or acceleration*) adj5 (step or stride or gait* or walk* or segment* or joint* or hip* or knee* or ankle* or torso* or center of mass or centre of mass or center of gravity or centre of gravity).mp.) OR exp biomechanics/

**Healthy Adult:** ((old* or young* or healthy or pain-free or able-bodied or asymptomatic or matched) adj5 (subject* or adult* or individual*)).mp. or exp control/ or exp control group/ or control*.mp.

**Validity/Reliability:** (valid* or reliab* or agreement*).mp. or exp validity/ or exp reliability/

**CINAHL:**

**Inertial Sensors:** (wearable sensor* or inertial sensor* or inertial measurement unit* or imu or acceleromet* or gyroscop* or magnetomet* or smart phone*)

**Gait Biomechanics:** ((speed* or time* or length* or width or cadence* or spatiotemporal or analys* or kinematic* or kinetic* or biomechanic* or angle* or acceleration*) N5 (step or stride or gait* or walk* or ambulat* or segment* or joint* or hip* or knee* or ankle* or torso* or “cent* of mass” or “cent* of gravity))

**Healthy Adults:** ((old* or young* or healthy or pain-free or able-bodied or asymptomatic or matched) adj5 (subject* or adult* or individual*) or (control group or control or controls))

**Validity/Reliability:** (valid* or reliab* or agreement*)

**Web of Science:**

**Inertial Sensors:** TS = (wearable* or smart phone* or inertial sensor* or inertial motion capture or inertial measurement unit* or imu or acceleromet* or gyroscop* or magnetomet*)

**Gait Biomechanics:** TS = ((speed or step time* or stride time* or step length* or stride length* or step width* or spatiotemporal or kinematic* or kinetic* or biomechanic* or analys* or (joint near/5 angle*) or (segment near/5 angle*) or (hip near/5 angle*) or (knee near/5 angle*) or (ankle near/5 angle*) or (foot near/5 angle*) or (acceleration* near/5 segment*) or (acceleration* near/5 torso*) or (acceleration* near/5 {centre of mass}) or (acceleration* near/5 {center of mass})) and (gait or walk* or ambulat*))

**Healthy Adults:** (((healthy NEAR/5 subject*) OR (healthy NEAR/5 adult*) OR (healthy NEAR/5 individual*) OR (young* NEAR/5 subject*) OR (young* NEAR/5 adult*) OR (young* NEAR/5 individual*) OR (old* NEAR/5 subject*) OR (old* NEAR/5 adult*) OR (old* NEAR/5 individual*) OR (pain-free NEAR/5 subject*) OR (pain-free NEAR/5 adult*) OR (pain-free NEAR/5 individual*) OR (able-bodied NEAR/5 subject*) OR (able-bodied NEAR/5 adult*) OR (able-bodied NEAR/5 individual*) OR (asymptomatic NEAR/5 subject*) OR (asymptomatic NEAR/5 adult*) OR (asymptomatic NEAR/5 individual*) OR (matched NEAR/5 subject*) OR (matched NEAR/5 adult*) OR (matched NEAR/5 individual*) OR control* OR {control group}))

**Validity/Reliability:** TS = (valid* or reliab* or agreement*)

**Compendex:**

**Inertial Sensors:** ((wearable sensor* or {smart phone*} or {inertial sensor*} or {inertial motion capture} or {inertial measurement unit*} or imu or acceleromet* or gyroscop* or magnetomet*) WN KY)

**Gait Biomechanics:** ((speed or step time* or stride time* or step length* or stride length* or step width* or spatiotemporal or kinematic* or kinetic* or biomechanic* or analys* or (joint near/5 angle*) or (segment near/5 angle*) or (hip near/5 angle*) or (knee near/5 angle*) or (ankle near/5 angle*) or (foot near/5 angle*) or (acceleration* near/5 segment*) or (acceleration* near/5 torso*) or (acceleration* near/5 {centre of mass}) or (acceleration* near/5 {center of mass})) WN KY) and ((gait or walk* or ambulat*) WN KY)

**Healthy Adults:** ((old* NEAR/5 individual*) or (old* near/5 subject*) or (old* near/5 adult*) or (young* NEAR/5 individual*) or (young* near/5 subject*) or (young* near/5 adult*) or (healthy NEAR/5 individual*) or (healthy near/5 subject*) or (healthy near/5 adult*) or (pain-free NEAR/5 individual*) or (pain-free near/5 subject*) or (pain-free near/5 adult*) or (able-bodied NEAR/5 individual*) or (able-bodied near/5 subject*) or (able-bodied near/5 adult*) or (asymptomatic NEAR/5 individual*) or (asymptomatic near/5 subject*) or (asymptomatic near/5 adult*) or (matched near/5 subject*) or (matched near/5 adult*) or {control} or {controls} or {control group}) WN KY

**Validity/Reliability:** ((valid* or reliab* or agreement*) WN KY)
