# Supplementary material for: Validity and reliability of wearable inertial sensors in healthy adult walking: a systematic review and meta-analysis
Source: J Neuroeng Rehabil. 2020 May 11;17:62. doi: 10.1186/s12984-020-00685-3 (PMC7216606; doi:10.1186/s12984-020-00685-3)
Supplement: Supplementary file 4 — Additional file 4 Supplementary Table 1. Qualitative summary of validity for spatiotemporal outcomes: r/ICC is presented as a weight average and range of reported values, while RMSE, Bias, and LOA widths are provided as the range of reported values. Gray shading identifies outcomes that have been quantitatively pooled in the results section. Supplementary Table 2. Qualitative summary of validity for other kinematic (and joint moment) outcomes: r/ICC is presented as a weight average and range of reported values, while RMSE, Bias, and LOA widths are provided as the range of reported values. Supplementary Table 3. Qualitative summary of validity for other biomechanical outcomes: r/ICC is presented as a weight average and range of reported values, while RMSE, Bias, and LOA widths are provided as the range of reported values. Supplementary Table 4. Qualitative summary of reliability for spatiotemporal outcomes: r/ICC is presented as a weight average and range of reported values, while SEM, MDC, Bias, and LOA widths are provided as the range of reported values. Supplementary Table 5. Qualitative summary of reliability for other kinematic outcomes: r/ICC is presented as a weight average and range of reported values, while SEM, MDC, Bias, and LOA widths are provided as the range of reported values. Supplementary Table 6. Qualitative summary of reliability for other biomechanical outcomes: r/ICC is presented as a weight average and range of reported values, while SEM, MDC, Bias, and LOA widths are provided as the range of reported values. [file 12984_2020_685_MOESM4_ESM.doc]

**Supplementary Table 1: Qualitative summary of validity for spatiotemporal outcomes: r/ICC is presented as a weight average and range of reported values, while RMSE, Bias, and LOA widths are provided as the range of reported values. Gray shading identifies outcomes that have been quantitatively pooled in the results section.**

| Outcome | Sensors/Placement | Studies | Quality | Total n | Results | | | | Interpretation |
| --- | --- | --- | --- | --- | --- | --- | --- | --- | --- |
|  |  |  |  |  | r/ICC | RMSE | Bias | LOA width |  |
| Step Time | Back | [28,51,61,88,91,93] | 32-77% | 76 | 1.00 | 15-30ms | 10ms | 6ms | Limited evidence for excellent validity |
|  | Shank/Ankle | [28,45,48,72,88,92] | 41-68% | 51 | 0.93 (0.89-0.97) | 12-28ms | -7-3ms | - | Limited evidence for good to excellent agreement |
|  | Multisensor | [89] | 58% | 24 | - | 20ms | 2ms | 60ms | Very limited evidence for moderate validity |
|  | Various placements (shoulder, purse, pocket, etc.) | [87] | 73% | 34 | 0.73 (0.60-0.89 | - | -17- -2ms | 103-218ms | Conflicting evidence |
| Stride Time | Multisensor | [23,24,81,89] | 46-68% | 53 | 0.96 | 10ms | -10-5ms | 40-162ms | Limited evidence for excellent validity |
|  | Back | [28,63,82,88,91,93,101] | 32-64% | 187 | 0.99 (0.99-0.99) | 28ms | 3-9ms | 4-12ms | Limited evidence for excellent validity |
|  | Shank/Ankle | [28,45,47,48,72,88,92,96] | 41-68% | 72 | 0.98 (0.98-0.99) | 7-18ms | -7-2ms | - | Limited evidence for excellent validity |
|  | Foot | [96] | 58% | 11 | 0.99 | - | - | - | Very limited evidence for excellent validity |
|  | Ear | [25,56] | 59-64% | 24 | - | - | - | - | Very limited evidence for moderate validity |
| Step Length | Multisensor | [22,34,66,89] | 41-72% | 56 | 0.87 (0.78-0.95) | 4-6.5cm | 0.3-0.6cm | 15-26cm | Limited evidence for good to excellent validity |
|  | Back | [51] | 77% | 23 | 0.99 | - | - | 15cm | Limited evidence for excellent validity |
|  | Various placements  (shoulder, pocket, etc.) | [61,86] | 32-72% | 43 | 0.71 (0.44-0.84) | 1.3cm | -13.4-0.6cm | 20-32cm | Conflicting evidence |
| Stride Length | Multisensor | [22,24,26,55,68,81,89] | 41-64% | 82 | 0.80 (0.80-0.84) | 4-11cm | -0.1-0.5cm | 14-45cm | Limited evidence for good validity |
|  | Back | [33] | 50% | 22 | 0.77 | - | - | - | Very limited evidence for good validity |
|  | Shank/ankle | [47,85,92,96] | 50-68% | 38 | 0.90 (0.89-0.92) |  | -12.6-2cm | - | Limited evidence for good to excellent validity |
|  | Foot | [59,85,96] | 32-58% | 21 | 0.99 | - | - | - | Very limited evidence for good to excellent validity |
| Stance Time | Multisensor | [24,80,89] | 46-58% | 45 | - | 20ms | -8-20ms | 60ms | Limited evidence for moderate validity |
|  | Back | [28,82,88,91,93] | 41-64% | 56 | - | 37ms | - | - | Limited evidence for moderate validity |
|  | Shank | [28,47,48,72,83,88,92,96] | 41-68% | 72 | 0.86 (0.33-0.99) | 18-37ms | -1-35ms | - | Conflicting evidence* |
|  | Foot | [96] | 58% | 11 | 0.95 | - | - | - | Very limited evidence for excellent validity |
|  | Ear | [56] | 64% | 10 | - | - | - | - | Very limited evidence for moderate validity |
| Swing Time | Multisensor | [80,89] | 54-58% | 36 | - | 20ms | - | 60ms | Very limited evidence for good validity |
|  | Back | [28,91,93] | 41-64% | 34 | - | 36ms | - | - | Very Limited evidence for moderate validity |
|  | Shank/Ankle | [28,47,48,72,92,96] | 41-68% | 52 | 0.77 (0.33-0.93) | 25-27ms | -58-25ms | - | Conflicting evidence* |
|  | Foot | [96] | 58% | 11 | 0.95 | - | - | - | Very limited evidence for excellent validity |
|  | Ear | [56] | 64% | 10 | - | - | - | - | Very limited evidence for moderate validity |
| Double Support | Multisensor | [23,26,80,81,89] | 54-68% | 68 | .957 | 20ms | -23-- 0.6ms | 70-78ms | Conflicting evidence |
|  | Back | [28,82,91] | 41-55% | 36 | - | 35ms | - | - | Very limited evidence for moderate validity |
|  | Shank | [28] | 41% | 10 | - | 28-30ms | - | - | Very limited evidence for moderate validity |
| Single Support | Multisensor | [23,68,82,89] | 54-68% | 47 | 0.87 | 20ms | 8-19ms | 60-145ms | Limited evidence for good validity |
|  | Back | [80] | 55% | 14 | - | - | - | - | Very limited evidence for moderate validity |
| Other phases (loading, flat foot, etc.) | Foot | [71] | 59% | 10 | - | - | - | - | Very limited evidence for moderate validity |
| Swing Width | Multisensor | [89] | 58% | 24 | - | 3 cm | -0.8 cm | 12 cm | Very limited evidence for moderate validity |
| Step Width | Multisensor | [89] | 58% | 24 | - | 3 cm | 0.8 cm | 11cm | Very limited evidence for moderate validity |
| Foot Clearance | Foot | [29,59,70] | 32-64% | 32 | - | 0.74 cm | - | - | Conflicting evidence |
| Step Time Variability | Back | [51,88] | 55-77% | 33 | 0.94 | - | - | 0.78% | Limited evidence for excellent validity |
|  | Ankle | [88] | 55% | 10 | - | - | - | - | Very limited evidence for moderate validity |
| Stride Time Variability | Multisensor | [81] | 55% | 9 | 0.70 | - | - | 2.7% | Very limited evidence for moderate validity |
|  | Back | [44,88] | 55-64% | 87 | 0.69 (0.53-0.85) | - | 1-2% | 6-12% | Very limited evidence for moderate to good validity |
|  | Shank/Ankle | [88] | 55% | 10 | - | - | - | - | Very limited evidence for moderate validity |
| Step Length Variability | Back | [51] | 77% | 23 | 0.33 | - | - | 3.5% | Limited evidence for poor validity |
| Stride Length Variability | Multisensor | [81] | 55% | 9 | 0.44 | - | - | 3% | Very limited evidence for poor validity |
| Step Time Symmetry | Ear | [25] | 59% | 14 | - | - | - | - | Very limited evidence for moderate validity |
| Stride Time Symmetry | Back | [44] | 64% | 77 | 0.11 (0.03-0.20) | - | 0ms | 5-6ms | Very limited evidence for poor validity |
| Stance Time Variability | Back | [88] | 55% | 10 | - | - | - | - | Very limited evidence for moderate validity |
|  | Ankle | [88] | 55% | 10 | - | - | - | - | Very limited evidence for moderate validity |

*Stance and swing time measure at the shank presented “Conflicting evidence” due to poor validity (r=0.33) reported by 1222. With 1222 removed, findings are “Limited evidence for excellent validity”.

Note: Three studies [30,40,69] examined the validity of spatiotemporal outcomes in healthy adults during walking, but only reported results combined together with clinical populations and/or altered gait.

**Supplementary Table 2: Qualitative summary of validity for other kinematic (and joint moment) outcomes: r/ICC is presented as a weight average and range of reported values, while RMSE, Bias, and LOA widths are provided as the range of reported values.**

| Outcome | Plane | Placement | Studies | Quality | Total n | Results | | | | Interpretation |
| --- | --- | --- | --- | --- | --- | --- | --- | --- | --- | --- |
|  |  |  |  |  |  | r/ICC | RMSE | Bias | LOA |  |
| Ankle Angle Waveform | Sagittal | Multisensor | [57,62,90,99] | 41%-67% | 49 | 0.80 (0.71-0.95) | 4.0°-7.8° | 2.1° | 10° | Limited evidence for moderate to excellent validity |
| Ankle Angle Discrete | Sagittal | Multisensor | [21,99] | 41%-83% | 35 | - | - | 0.4° | - | Very limited evidence for good validity |
| Ankle Angle Waveform | Frontal | Multisensor | [57,90,99] | 41%-67% | 49 | 0.79 | 2.97-9.7° | 1.81° | 3.1° | Limited evidence for good validity |
| Ankle Angle Discrete | Frontal | Multisensor | [21,99] | 41%-83% | 35 | - | - | 1.38° | - | Very limited evidence for good validity |
| Ankle Angle Waveform | Transverse | Multisensor | [90,99] | 41%-67% | 38 | - | 3.6° | 1.8° | 5.8° | Very limited evidence for good validity |
| Ankle Angle Discrete | Transverse | Multisensor | [21,99] | 41%-83% | 35 | - | - | 1.3° | - | Very limited evidence for good validity |
| Knee Angle Waveform | Sagittal | Multisensor | [39,57,62,90,99] | 36%-67% | 66 | 0.98 | 0.7°-4.6° | - | 5.1° | Limited evidence for excellent validity |
| Knee Angle Discrete | Sagittal | Multisensor | [21,99] | 41%-83% | 35 | - | - | 0.8° | - | Very limited evidence for excellent validity |
| Knee Angle Waveform | Frontal | Multisensor | [75,90,99] | 41%-67% | 44 | - | 3.8°-11.2° | 5.1° | 9.3° | Very limited evidence for moderate validity |
| Knee Angle Discrete | Frontal | Multisensor | [21,36,99] | 41%-83% | 37 | - | - | 1.5° | - | Very limited evidence for good validity |
| Knee Angle Waveform | Transverse | Multisensor | [90,99] | 41%-67% | 38 | - | 4.5° | 2.7° | 10.2° | Very limited evidence for moderate validity |
| Knee Angle Discrete | Transverse | Multisensor | [21,99] | 41%-83% | 35 | - | - | 0.0° | - | Very limited evidence for excellent validity |
| Hip Angle Waveform | Sagittal | Multisensor | [57,62,75,90] | 41%-67% | 65 | 0.97 (0.95-0.99) | 2.7°-6.3° | 1.5° | 3.3° | Limited evidence for excellent validity |
| Hip Angle Discrete | Sagittal | Multisensor | [21,99] | 41%-83% | 35 | - | - | 2.4°-2.5° | - | Very limited evidence for good validity |
| Hip Angle Waveform | Frontal | Multisensor | [57,90,99] | 41%-67% | 49 | 0.91 | 2.6°-3.9° | 4.8° | 6° | Limited evidence for good to excellent validity |
| Hip Angle Discrete | Frontal | Multisensor | [21,99] | 41%-83% | 35 | - | - | 5.4° | - | Very limited evidence for moderate validity |
| Hip Angle Waveform | Transverse | Multisensor | [57,90] | 64%-67% | 39 | 0.66 | 5.0°-6.5° |  | 10.1° | Limited evidence for moderate validity |
| Hip Angle Discrete | Transverse | Multisensor | [21,99] | 41%-83% | 35 | - | - | 0.0°-3.0° | - | Very limited evidence for excellent validity |
| Foot Segment Angle Waveform | Sagittal | Multisensor | [35,62] | 27%-59% | 21 | 1.0* | 2.1°-4.9° | - | - | Very limited evidence for excellent validity |
| Foot Segment Angle Waveform | Transverse | Foot | [54] | 59% | 13 | - | 1.8° | - | - | Very limited evidence for excellent validity |
| Foot Segment Angle Discrete | Transverse | Foot | [98] | 59% | 14 | 1.0 | - | 0.1° | - | Very limited evidence for excellent validity |
| Shank Segment Angle Waveform | Sagittal | Sibia | [62] | 59% | 20 | 1.0 | 2.4° | - | - | Very limited evidence for excellent validity |
| Shank Segment Angle Discrete | Sagittal | Shank | [24] | 45% | 9 | - | - | -0.3 | - | Very limited evidence for excellent validity |
| Thigh Segment Angle Waveform | Sagittal | Thigh | [62,65] | 45%-59% | 28 | 0.97 (0.94-0.98) | 1.6°-4.1° | - | - | Very limited evidence for excellent validity |
| Thigh Segment Angle Waveform | Frontal | Thigh | [65] | 0.454545455 | 8 | 0.90 | 3.5° | - | - | Very limited evidence for excellent validity |
| Thigh Segment Angle Discrete | Sagittal | Thigh | [20,24] | 32%-45% | 28 | 1.0 (0.97-1.0) | 1.8°-2.5° | 2.9° | - | Very limited evidence for excellent validity |
| Pelvis Angle Waveform | Sagittal | Back | [31,62,90] | 59%-73% | 65 | 0.89 (0.84-0.94) | 1.8°-2.7° | - | 1.6° | Limited evidence for good validity |
| Pelvis Angle Waveform | Frontal | Back | [31,90] | 67-73% | 45 | 0.91 | 2.6-2.7° | - | 4.0° | Limited evidence for excellent validity |
| Pelvis Angle Waveform | Transverse | Back | [90] | 67% | 28 | - | - | - | 1.7° | Very limited evidence for good validity |
| Ankle Joint Moment Waveform | Sagittal | Multisensor | [57] | 64% | 11 | 0.94 | 1.5** | - | - | Very limited evidence for excellent validity |
| Ankle Joint Moment Waveform | Frontal | Multisensor | [57] | 64% | 11 | 0.75 | 0.6** | - | - | Very limited evidence for good validity |
| Ankle Joint Moment Waveform | Transverse | Multisensor | [57] | 64% | 11 | 0.7 | 0.5** | - | - | Very limited evidence for moderate validity |
| Knee Joint Moment Waveform | Sagittal | Multisensor | [57,75] | 41%-64% | 17 | 0.59 | 0.8-2.2** | - | - | Very limited evidence for moderate validity |
| Knee Joint Moment Waveform | Frontal | Multisensor | [57] | 64% | 11 | 0.83 | 1.3** | - | - | Very limited evidence for good validity |
| Knee Joint Moment Waveform | Transverse | Multisensor | [57] | 64% | 11 | 0.73 | 0.5** | - | - | Very limited evidence for moderate validity |
| Thigh Joint Moment Waveform | Sagittal | Multisensor | [57,75] | 41%-64% | 17 | 0.92 | 0.6-1.8** | - | - | Very limited evidence for excellent validity |
| Thigh Joint Moment Waveform | Frontal | Multisensor | [57] | 64% | 11 | 0.84 | 1.0** | - | - | Very limited evidence for good validity |
| Thigh Joint Moment Waveform | Transverse | Multisensor | [57] | 64% | 11 | 0.47 | 0.3** | - | - | Very limited evidence for poor validity |

**RMSE values were reported as %BW*ht or Nm/BW (BW, body weight; ht, height)

**Supplementary Table 3: Qualitative summary of validity for other biomechanical outcomes: r/ICC is presented as a weight average and range of reported values, while RMSE, Bias, and LOA widths are provided as the range of reported values.**

| Outcome | Placement | Studies | Quality | Total n | Results | | | | Interpretation |
| --- | --- | --- | --- | --- | --- | --- | --- | --- | --- |
|  |  |  |  |  | r/ICC | RMSE | Bias | LOA width |  |
| Max Lyapunov (long) | Back | [32] | 55% | 9 | 3D = 0.99 | - | - | - | Very limited evidence for excellent agreement |
| Max Lyapunov (short) | Back | [32] | 55% | 9 | 3D = 0.88 | - | - | - | Very limited evidence for good agreement |
| Max Floquet multiplier | Back | [32] | 55% | 9 | 3D = 0.66 | - | - | - | Very limited evidence for moderate agreement |
| Accelerations (discrete peaks) | Back | [42,87,97] | 36-64% | 54 | V = 0.67 (0.53-0.95) | - | RES = 0g;  V = 0.6g | RES = 0.11g;  V = 0.42g | Conflicting evidence |
| Accelerations (waveform RMS) | Torso | [38] | 68% | 48 | AP = 0.86 (0.84-0.88);  ML = 0.74 (0.63-.84);  V = 0.98 (0.98-0.98) | - | AP = -0.01-0g;  ML = 0-0.01g;  V = 0g | - | Very limited evidence for moderate, good, and excellent validity in ML, AP, and V directions, respectively |
| GRF | Multisensor | [75] | 41% | 6 | - | Horz = 0.56N/BW;  V = 0.27N/BW | - | - | Conflicting evidence |
| COM displacement | Back | [42,43,63,79,87] | 32-68% | 61 | V = 0.52 (0.21-0.78) | - | - | AP=1.6cm; ML=1.8cm; V=0.8-2.5cm | Conflicting evidence |

Abbreviations: vertical = V, mediolateral = ML, anteroposterior = AP, resultant = RES; Horz = Horizontal (ML/AP)

**Supplementary Table 4: Qualitative summary of reliability for spatiotemporal outcomes: r/ICC is presented as a weight average and range of reported values, while SEM, MDC, Bias, and LOA widths are provided as the range of reported values.**

| Outcome | Placement | Studies | Quality | Total n | Results | | | | | Interpretation |
| --- | --- | --- | --- | --- | --- | --- | --- | --- | --- | --- |
|  |  |  |  |  | r/ICC | SEM | MDC | Bias | LOA width |  |
| Step Time | Multisensor | [46,89] | 58-75% | 42 | 0.92 (0.87-0.98) | - | - | - | - | Limited evidence for good to excellent reliability |
|  | Back | [52] | 75% | 23 | 0.91 | - | - | - | 60ms | Limited evidence for excellent reliability |
|  | Foot | [96] | 58% | 33 | 0.98 (0.96-0.99) | - | 30-60ms | - | - | Very limited evidence for excellent reliability |
| Stride Time | Multisensor | [76,89] | 58-75% | 39 | 0.91 (0.90-0.92) | 40ms | - | 10ms | 111ms | Limited evidence for excellent reliability |
|  | Ankle | [96] | 58% | 33 | 0.98 (0.98-0.99) | - | 40-120ms | - | - | Very limited evidence for excellent reliability |
| Step Length | Multisensor | [46,89] | 58-75% | 42 | 0.76 (0.67-0.88) | - | - | - | - | Limited evidence for moderate to good reliability |
|  | Back | [52,53,100] | 63-75% | 63 | 0.95 (0.95-0.95) | - | - | - | 8cm | Limited evidence for excellent reliability |
| Stride Length | Multisensor | [64,76,80,89] | 54-75% | 71 | 0.82 (0.76-0.88) | - | - | 10cm | 58cm | Limited evidence for good reliability |
|  | Back | [53] | 67% | 20 | 0.94 | - | - | 0cm | - | Very limited evidence for excellent reliability |
|  | Ankle | [96] | 58% | 33 | 0.96 (0.95-0.97) | - | 70-80cm | - | - | Very limited evidence for excellent reliability |
| Stance Time | Multisensor | [76,80,89] | 54-75% | 61 | 0.90 (0.89-0.92) | - | - | 6ms | 109ms | Limited evidence for excellent reliability |
|  | Ankle | [96] | 58% | 33 | 0.81 (0.65-0.92) | - | 2.8-3.4% | - | - | Limited evidence for good reliability |
| Swing Time | Multisensor | [46,76,80,89] | 54-75% | 79 | 0.80 (0.73-0.97) | - | - | - | 62ms | Limited evidence for good reliability |
|  | Ankle | [96] | 58% | 33 | 0.81 (0.65-0.92) | - | 2.8-3.4% | - | - | Limited evidence for good reliability |
| Double Support | Multisensor | [80,89] | 54-58% | 36 | 0.90 | - | - | - | - | Very limited evidence for excellent reliability |
| Single Support | Multisensor | [80,89] | 54-58% | 36 | 0.85 | - | - | - | - | Very limited evidence for good reliability |
| Swing Width | Multisensor | [89] | 58% | 24 | 0.69 | - | - | - | - | Very limited evidence for moderate reliability |
| Step Width | Multisensor | [89] | 58% | 24 | 0.25 | - | - | - | - | Very limited evidence for poor reliability |
| Foot Clearance | Multisensor | [76] | 75% | 25 | 0.85 | 21°/s | - | 0.78°/s | 60°/s | Limited evidence for good reliability |
|  | Foot | [49] | 50% | 19 | 0.85 | - | - | -.1-0cm | 1-1.2cm | Very limited evidence for good reliability |
| Step Time Variability | Back | [52] | 75% | 23 | 0.78 | - | - | - | 0.89% | Limited evidence for good reliability |
| Step Length Variability | Back | [52] | 75% | 23 | 0.31 | - | - | - | 4.10% | Limited evidence for poor reliability |
| Stride Length Variability | Foot | [49] | 50% | 19 | 0.52 | - | - | -0.14-0.57% | 1.66-2.08% | Very limited evidence for moderate reliability |
| Step Time Symmetry | Back | [27] | 75% | 81 | 0.82 (0.80-0.88) | - | 2.4-2.8% | 0.1-0.8% | - | Limited evidence for good reliability |

Note: One study [69] examined stride time reliability in healthy adults, but only reported results combined with altered gait.

**Supplementary Table 5: Qualitative summary of reliability for other kinematic outcomes: r/ICC is presented as a weight average and range of reported values, while SEM, MDC, Bias, and LOA widths are provided as the range of reported values.**

| Outcome | Plane | Placement | Studies | Quality | Total n | Results | | | | | Interpretation |
| --- | --- | --- | --- | --- | --- | --- | --- | --- | --- | --- | --- |
|  |  |  |  |  |  | r/ICC | SEM | MDC | Bias | LOA |  |
| Ankle Angle Waveform | Sagittal | Multisensor | [90] | 67% | 28 | 0.95 | - | - | - | - | Very limited evidence for excellent reliability |
| Ankle Angle Discrete | Sagittal | Multisensor | [21,94] | 75%-83% | 64 | 0.92 (0.9-0.94) | 1.8°-2.9° | 4.9°-8.0° | 0°-0.6° | - | Moderate quality evidence for excellent reliability |
| Ankle Angle Waveform | Frontal | Multisensor | [90] | 67% | 28 | 0.77 | - | - | - | - | Very limited evidence for good reliability |
| Ankle Angle Discrete | Frontal | Multisensor | [21,94] | 75%-83% | 44 | 0.86 (0.76-0.92) | 1.5°-2.4° | 4.2°-6.7° | 0.1°-0.8° | - | Moderate quality evidence for good reliability |
| Ankle Angle Waveform | Transverse | Multisensor | [90] | 67% | 28 | 0.86 | - | - | - | - | Very limited evidence for good reliability |
| Ankle Angle Discrete | Transverse | Multisensor | [21,94] | 75%-83% | 44 | 0.81 (0.7-0.9) | 3.3°-3.8° | 9.2°-10.6° | 0°-1.2° | - | Moderate quality evidence for good reliability |
| Knee Angle Waveform | Sagittal | Multisensor | [90] | 67% | 28 | 0.98 | - | - | - | - | Very limited evidence for excellent reliability |
| Knee Angle Discrete | Sagittal | Multisensor | [21,94] | 75%-83% | 24 | 0.91 (0.88-0.93) | 1.9°-2.4° | 5.2°-6.6° | - | - | Moderate quality evidence for excellent reliability |
| Knee Angle Waveform | Frontal | Multisensor | [90] | 67% | 28 | 0.56 | - | - | - | - | Very limited evidence for moderate reliability |
| Knee Angle Discrete | Frontal | Multisensor | [21,94] | 75%-83% | 44 | 0.46 (0.08-0.71) | 2.0°-3.9° | 5.6°-10.9° | - | - | Conflicting evidence |
| Knee Angle Waveform | Transverse | Multisensor | [90] | 67% | 20 | 0.69 | - | - | - | - | Very limited evidence for moderate reliability |
| Knee Angle Discrete | Transverse | Multisensor | [21,94] | 75%-83% | 44 | 0.84 (0.8-0.88) | 3.0°-3.4° | 8.3°-9.4° | - | - | Moderate quality evidence for good reliability |
| Hip Angle Waveform | Sagittal | Multisensor | [90] | 67% | 28 | 0.98 | - | - | - | - | Very limited evidence for excellent reliability |
| Hip Angle Discrete | Sagittal | Multisensor | [21,94] | 75%-83% | 44 | 0.64 (0.57-0.77) | 3.2°-3.6° | 8.8°-9.9° | 0.2°-0.7° | - | Moderate quality evidence for moderate reliability |
| Hip Angle Waveform | Frontal | Multisensor | [90] | 67% | 28 | 0.92 | - | - | - | - | Very limited evidence for excellent reliability |
| Hip Angle Discrete | Frontal | Multisensor | [21,94] | 75%-83% | 44 | 0.52 (0.49-0.62) | 1.9-2.5 | 5.4-6.1 | - | - | Moderate quality evidence for moderate reliability |
| Hip Angle Waveform | Transverse | Multisensor | [90] | 67% | 28 | 0.76 | - | - | - | - | Very limited evidence for good reliability |
| Hip Angle Discrete | Transverse | Multisensor | [21,94] | 75%-83% | 44 | 0.0 (0.0-0.02) | 0.8°-6.7° | 2.2°-18.7° | 0.2°-0.7° | - | Moderate quality evidence for poor reliability |
| Foot Angle Discrete | Transverse | Foot | [37] | 88% | 20 | 0.95 | 1.3° | 3.5° | 0.1° | - | Very limited evidence for excellent reliability |
| Pelvis Angle Waveform | Sagittal | Back | [76,90] | 67%-75% | 53 | 0.84 (0.77-0.90) | 1.7° | - | 0.43° | 4.9° | Very limited evidence for good reliability |
| Pelvis Angle Discrete | Sagittal | Back | [94] | 75% | 20 | 0.74 (0.62-0.82) | - | - | - | - | Very limited evidence for moderate reliability |
| Pelvis Angle Waveform | Frontal | Back | [76,90] | 67%-75% | 53 | 0.71 (0.52-0.92) | 1.7° | - | 0.40° | 4.7° | Very limited evidence for moderate reliability |
| Pelvis Angle Discrete | Frontal | Back | [94] | 75% | 20 | 0.62 (0.48-0.68) | - | - | - | - | Very limited evidence for moderate reliability |
| Pelvis Angle Waveform | Transverse | Back | [76,90] | 67%-75% | 53 | 0.89 (0.81-0.98) | 2.2° | - | 0.15° | 6.2° | Very limited evidence for good reliability |
| Pelvis Angle Discrete | Transverse | Back | [94] | 75% | 20 | 0.51 (0.48-0.57) | - | - | - | - | Very limited evidence for moderate reliability |

**Supplementary Table 6: Qualitative summary of reliability for other biomechanical outcomes: r/ICC is presented as a weight average and range of reported values, while SEM, MDC, Bias, and LOA widths are provided as the range of reported values.**

| Outcome | Placement | Studies | Quality | Total n | Results | | | | | Interpretation |
| --- | --- | --- | --- | --- | --- | --- | --- | --- | --- | --- |
|  |  |  |  |  | r/ICC | SEM | MDC | Bias | LOA |  |
| Max Lyapunov (short – 0.5-1 stride) | Trunk | [50,78,95] | 58-71% | 154 | AP=0.53 (0.51-0.55);  V=0.67 (0.66-0.6);  3D=0.56 (0.52-0.63) | AP=0.06-0.09;  V=0.06-0.1 | AP=23-33;  V=21-28 | - | - | Very limited evidence for moderate reliability  **ML results quantitatively pooled* |
|  | Foot | [50] | 58% | 39 | AP=0.80;  ML=0.64;  3D=0.44 | - | - | - | - | Very limited evidence for poor, moderate, and good reliability in 3D, ML, and AP directions, respectively |
| Max Lyapunov (long – 4-10 strides) | Trunk | [78] | 71% | 95 | AP=0.47;  ML=0.60;  V=0.67 | 0.01 | AP=107;  ML=0.76;  V=67 | - | - | Limited evidence for poor to moderate reliability |
| Accelerations (RMS of waveform) | Back | [53,58,73,74] | 50-79% | 77 | AP=0.91 (0.89-93);  ML=0.83 (0.82-0.92);  V=0.88 (0.78-0.90);  RES=0.87 (0.85-0.89) | - | - | - | - | Limited evidence for good reliability in ML, V, and RES directions, but excellent reliability in AP |
|  | Shank | [58,67] | 67-79% | 17 | AP=0.97;  ML=0.92;  V=0.97 (0.96-0.97);  RES=0.71 | - | - | - | - | Limited evidence for excellent reliability in AP, ML, and V directions, but moderate in RES |
| Step Symmetry | Back | [27] | 75% | 81 | ML=0.72 (0.63-0.81);  V=0.70 (0.58-0.83) | 0.09-0.1 | - | - | - | Limited evidence for moderate reliability |
| Stride Regularity | Back | [27,74] | 50-75% | 111 | ML=0.79 (0.71-0.87);  V=0.79 (0.70-0.91) | 0.07-0.1 | - | - | - | Limited evidence for good reliability |
| Peak Frequency | Back | [74] | 50% | 30 | 0.91 | - | - | - | - | Very limited evidence for excellent reliability |
| Coefficient of Variance | Back | [74] | 50% | 30 | 0.78 | - | - |  |  | Very limited evidence for good reliability |
| Vertical Displacement | Back | [84] | 54% | 24 | 0.53 | - | - | - | - | Very limited evidence for moderate reliability |

Abbreviations: vertical = V, mediolateral = ML, anteroposterior = AP, resultant = RES
